# Recent Invasion of the Symbiont-Bearing Foraminifera *Pararotalia* into the Eastern Mediterranean Facilitated by the Ongoing Warming Trend

**DOI:** 10.1371/journal.pone.0132917

**Published:** 2015-08-13

**Authors:** Christiane Schmidt, Raphael Morard, Ahuva Almogi-Labin, Anna E. Weinmann, Danna Titelboim, Sigal Abramovich, Michal Kucera

**Affiliations:** 1 MARUM, Center for Marine Environmental Sciences, University of Bremen, Bremen, Germany; 2 Geological Survey of Israel, Jerusalem, Israel; 3 Department of Geology, University of Georgia, Athens, Georgia, United States of America; 4 Steinmann-Institute for Geology, Mineralogy und Paleontology, University of Bonn, Bonn, Germany; 5 Department of Geological and Environmental Sciences, Ben Gurion University of the Negev, Beer-Sheva, Israel; Università di Genova, ITALY

## Abstract

The eastern Mediterranean is a hotspot of biological invasions. Numerous species of Indo-pacific origin have colonized the Mediterranean in recent times, including tropical symbiont-bearing foraminifera. Among these is the species *Pararotalia calcariformata*. Unlike other invasive foraminifera, this species was discovered only two decades ago and is restricted to the eastern Mediterranean coast. Combining ecological, genetic and physiological observations, we attempt to explain the recent invasion of this species in the Mediterranean Sea. Using morphological and genetic data, we confirm the species attribution to *P*. *calcariformata* McCulloch 1977 and identify its symbionts as a consortium of diatom species dominated by *Minutocellus polymorphus*. We document photosynthetic activity of its endosymbionts using Pulse Amplitude Modulated Fluorometry and test the effects of elevated temperatures on growth rates of asexual offspring. The culturing of asexual offspring for 120 days shows a 30-day period of rapid growth followed by a period of slower growth. A subsequent 48-day temperature sensitivity experiment indicates a similar developmental pathway and high growth rate at 28°C, whereas an almost complete inhibition of growth was observed at 20°C and 35°C. This indicates that the offspring of this species may have lower tolerance to cold temperatures than what would be expected for species native to the Mediterranean. We expand this hypothesis by applying a Species Distribution Model (SDM) based on modern occurrences in the Mediterranean using three environmental variables: irradiance, turbidity and yearly minimum temperature. The model reproduces the observed restricted distribution and indicates that the range of the species will drastically expand westwards under future global change scenarios. We conclude that *P*. *calcariformata* established a population in the Levant because of the recent warming in the region. In line with observations from other groups of organisms, our results indicate that continued warming of the eastern Mediterranean will facilitate the invasion of more tropical marine taxa into the Mediterranean, disturbing local biodiversity and ecosystem structure.

## Introduction

Human activities can induce invasions of marine species in two ways: indirectly, by altering the climate and ecosystems [1], facilitating range expansions [2, 3], or directly, by mediating species dispersal through anthropogenic means [4]. The latter can be realized either as active transport, such as ship traffic, or as removal of barriers to natural dispersal. The ongoing anthropogenic global change is altering ecosystems at a faster rate than seen in the recent geological past. In consequence, many species are unable to adapt to locally changing conditions through phenotypic plasticity and evolutionary processes and respond by shifting their geographical ranges [5, 6]. This is particularly relevant for marine ecosystems, where species appear to spread an order of magnitude faster than in the terrestrial realm [2]. Temperature is the key variable controlling the spread of species and can be used to predict biogeographic range expansions in shallow marine communities [7].

An excellent example of directly mediated invasion of marine species is the opening of the Suez Canal. This event facilitated what is known as the Lessepsian invasion, describing the movement of species from the Red Sea into the Mediterranean after the opening of the Canal in 1869 [8, 9]. If the opening of the Suez Canal was the only factor needed to trigger the range extension of Indopacific species into the Mediterranean, thus all Lessepsian migrants should have appeared near simultaneously. However, the eastern Mediterranean has been experiencing a strong warming trend over the last 20 years [10] altering the environmental conditions at the exit of the Lessepsian corridor. The expansion of Indo-pacific species into the Mediterranean is thus likely exacerbated by climate change and many of the migrating marine species including fish, algae, plants and invertebrates are continuing to expand their range [6]. As a result, the Levantine ecosystem is already dominated by non-native fish species leading to a significant decline of the indigenous populations [11].

A particularly successful group among the Lessepsian migrants are symbiont-bearing benthic foraminifera [12, 13]. The passive dispersal of these organisms appears to be facilitated through the transport of propagules, which include asexually and sexually reproduced offspring [14, 15]. Alternatively, like in many other Lessepsian migrants, the association with marine macroalgae [9, 16] or fish [17] represents another means of passive dispersal of benthic foraminifera into the Mediterranean. Also the introduction of foraminiferal species in new habitats via ballast waters has been documented [18]. The establishment of invasive populations is possible in a new habitat as long as the local temperature regime facilitates the completion of the full reproductive cycle of the invasive species [19]. Symbiont-bearing foraminifera have well defined, species-specific temperature tolerances [20]. Temperatures exceeding the upper thermal threshold cause symbiont bleaching [21], whilst low temperatures prevent the establishment of populations [20]. The symbiosis in benthic foraminifera provides an energetic advantage [22], with the photosymbiont having a double role, by providing nutrition [23] and promoting calcification [24]. Symbiont-bearing foraminifera are ideally adapted to the oligotrophic conditions of the eastern Mediterranean [25]. Indeed, the appearance of invasive symbiont-bearing foraminifera in the eastern Mediterranean is documented at least since the 1960s [26] and their ongoing proliferation has significant impact on coastal ecosystems [27].

The most recently described migration of symbiont-bearing benthic foraminifera into the Levantine basin involves a species of the Indopacific genus *Pararotalia*. This species has been first reported in the Levant in 1994 [28, 29]. It has since then been found to proliferate along the Mediterranean coast from Israel [30, 31] to southern Turkey [32]. The modern foraminiferal fauna of the Mediterranean Sea is mostly of Atlantic origin [26, 33]. After the opening of the Suez Canal in 1869, many tropical symbiont-bearing foraminifera migrated into the Mediterranean Sea, including amphisteginids, soritids, and heterosteginids [34]. The apparently later invasion and more restricted occurrence of *Pararotalia* contrasts with other symbiont-bearing foraminifera, implying that the invasion of *Pararotalia* was not facilitated solely by the physical connection of the Suez Canal. We note that the invasive *Pararotalia* is not a “classical” Lessepsian species, as it has not yet been found in the Red Sea [35–37]. However, with the exception of the Gulf of Aqaba [38], the diversity of foraminifera in the Red Sea is not well known and considering the habitat of the species and its distribution in the Indopacific, it is likely that it also occurs in the Red Sea [39].

To understand the explosive recent invasion of *Pararotalia* in the Mediterranean, we carried out an investigation of the ecology and physiology of this species. We investigated its genetic relationship with Pacific populations of the genus, identified its endosymbiotic microalgae, determined its photosynthetic activity from freshly collected specimens and monitored their photosynthetic activity in the laboratory cultures. We hypothesized that like in other foraminifera [40] and in many other marine species [7] temperature is the main factor controlling the establishment of new populations. Therefore, we carried out an experiment exposing asexual offspring of *P*. *calcariformata* originating from the invasive population in the Mediterranean to three temperatures (20°C, 28°C and 35°C) to determine their survival and growth rates under these conditions. Using a compilation of all occurrence records of the species in the Mediterranean, we model its likely spread under future climate change.

## Results and Discussion

### The identity of the invasive species and its current distribution

*Pararotalia calcariformata* McCulloch 1977 has been only very recently added to the list of over 700 marine species that appear to have invaded the Mediterranean in historical times [9]. The species has been reported from littoral environments of the Israeli coast as *Eponides* [29] and as *Pararotalia spinigera* [28]. The earliest description in the Levant of the genus *Pararotalia* is by [41], who reported in 1961 the occurrence of *Pararotalia* cf. *ozawai* (Graham & Millitante non Asano) from a locality near Haifa. Unfortunately, the study by [41] provides no illustration and the material of the collection is not available. Therefore, we cannot confirm this record and conclude that the exact time of introduction of the species cannot be constrained beyond occurring prior to 1994. However, we note that the species must be a very recent invader, as specimens attributed to *P*. *calcariformata* were never found in historical layers, and are only present in surface sediments [42]. All fossil occurrences of the genus *Pararotalia* in the Mediterranean are pre-Quaternary (see S1 File) [43]. Other studies confirmed the proliferation of the species *P*. *calcariformata* after 1994, identifying large populations ranging from the southernmost Israeli coast [30, 31] to southern Turkey [32]. All localities where this species has been reported up to now in the literature as well as through our investigations in the eastern Mediterranean are given in S1 Table.

Considering the large abundance of the species and its distinctive shape, it is unlikely that it has been overlooked in earlier studies or not seen at other localities in the eastern Mediterranean [12, 44]. Collectively, the existing evidence implies that the species has established a population in the Levant only recently and began to proliferate and expand its range within the last two decades [32]. The majority of the invasive species in the eastern Mediterranean represent Lessepsian migrations through the Suez Canal [9], which migrated through the Canal since its opening in 1869, as it was the first artificial connection between Europe and Asia. More recently migrations were facilitated by several changes of the Canal’s width and depth, the most recent in 2010. *Pararotalia* has not yet been described from potential source locations in the Red Sea such as the Gulf of Aqaba [35], the Gulf of Suez [37], the Bay of Safaga [45] or the South Sinai Coast [36]. On the other hand, *Pararotalia* appears to be an Indopacific genus [20] and specimens assignable to *P*. *calcariformata* have been found along the coast of the Arabian Sea and Oman (S1 Table). Therefore, it is most likely that *P*. *calcariformata* also followed the Lessepsian route. Under this scenario, the species could have invaded the Mediterranean across the Suez Canal. The genus *Pararotalia* has been shown to travel attached to gastropod larvae [46] or inside guts of fish [17]. Alternatively, it could have been introduced with ballast waters and sediment, as has been hypothesized for the red alga *Grateloupia yinggehaiensis* possibly introduced to the Mediterranean from China by ship traffic [47]. This alga appears to have established a continuous population in the vicinity of a thermoelectric power plant [47]. We note that *P*. *calcariformata* has been found to occur prolifically in the heat plume of the thermal power plant in Hadera [31], which might also has served as a stepping stone for its invasion. The exact origin of *P*. *calcariformata* and its dispersal route can only be established by the identification of a potential parent population, such as has been shown for the invasive *Sorites* by [13], who found a potential parent population in the Gulf of Aqaba. Therefore, we conducted a taxonomical and genetic investigation to characterize the relationship between the Indopacific and Levantine populations of *Pararotalia*.

**Fig 1:**
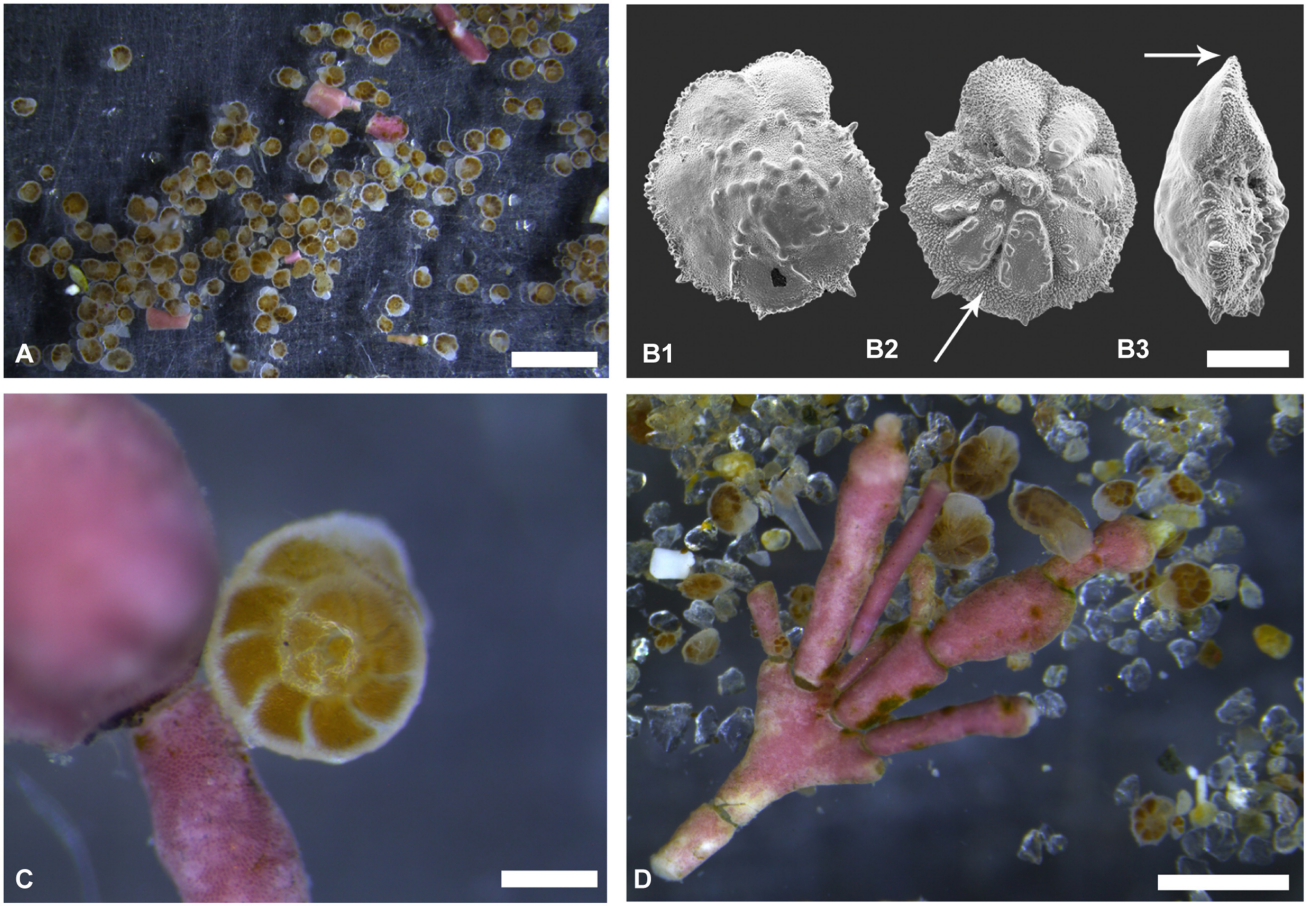
Shell morphology and live appearance of the foraminifer *Pararotalia calcariformata* collected from Nachsholim National Park, Israel. A) Living specimens picked from algae substrate, scale bar 2 mm. B) Representative specimen in spiral, umbilical and lateral views, images taken with scanning electron microscopy, in image B2 arrows indicate septal interlocular spaces and in image B3 the keel of the foraminifer, special features supporting the identification of the species in line with the original description by McCulloch 1977, scale bar 100 μm. C, D) Living specimens attached on their substrate, the coralline algae *Jania rubens*, scale bar 200 μm in C), and 1 mm in D).

The morphological taxonomy of the invasive *Pararotalia* population has been confusing, because the first specimens found and identified in the Mediterranean were mistakenly classified as *Eponides repandus* (Fichtel and Moll, 1798) [29] and only later assigned to *Pararotalia spinigera* Le Calvez 1949 [48, 49]. The generic classification has been stable since, but the species level taxonomy within this morphologically variable genus is in need of revision. Previous studies [28, 30, 31] designated recent specimens from the Israeli coast as *Pararotalia spinigera* Le Calvez 1949, following [50, 51]. The study by [32] first suggested that the specimens may rather represent *P*. *calcariformata*, an extant species described from the Indian Ocean by [52]. *Pararotalia calcariformata* McCulloch 1977 is mainly distinguished from *P*. *spinigera* Le Calvez 1949 as described by [50, 51] in having a distinct peripheral keel and deep septal interlocular spaces on the umbilical side (Fig 1B2 and 1B3). A comparison of the morphology of the Levantine species with this description confirms that the assignment of the invasive species to *Pararotalia spinigera* is incorrect, because a keel and deep septal interlocular space are clearly present among the Levantine specimens. Our analysis also confirms that the specimens we examined have similar appearance as those reported by [32] from Hatay, Turkey. Therefore, we here conclude that morphologically, the invasive species shows most affinity with the concept of *P*. *calcariformata* and we use this name henceforth when referring to the extant populations from the Levant.

**Fig 2:**
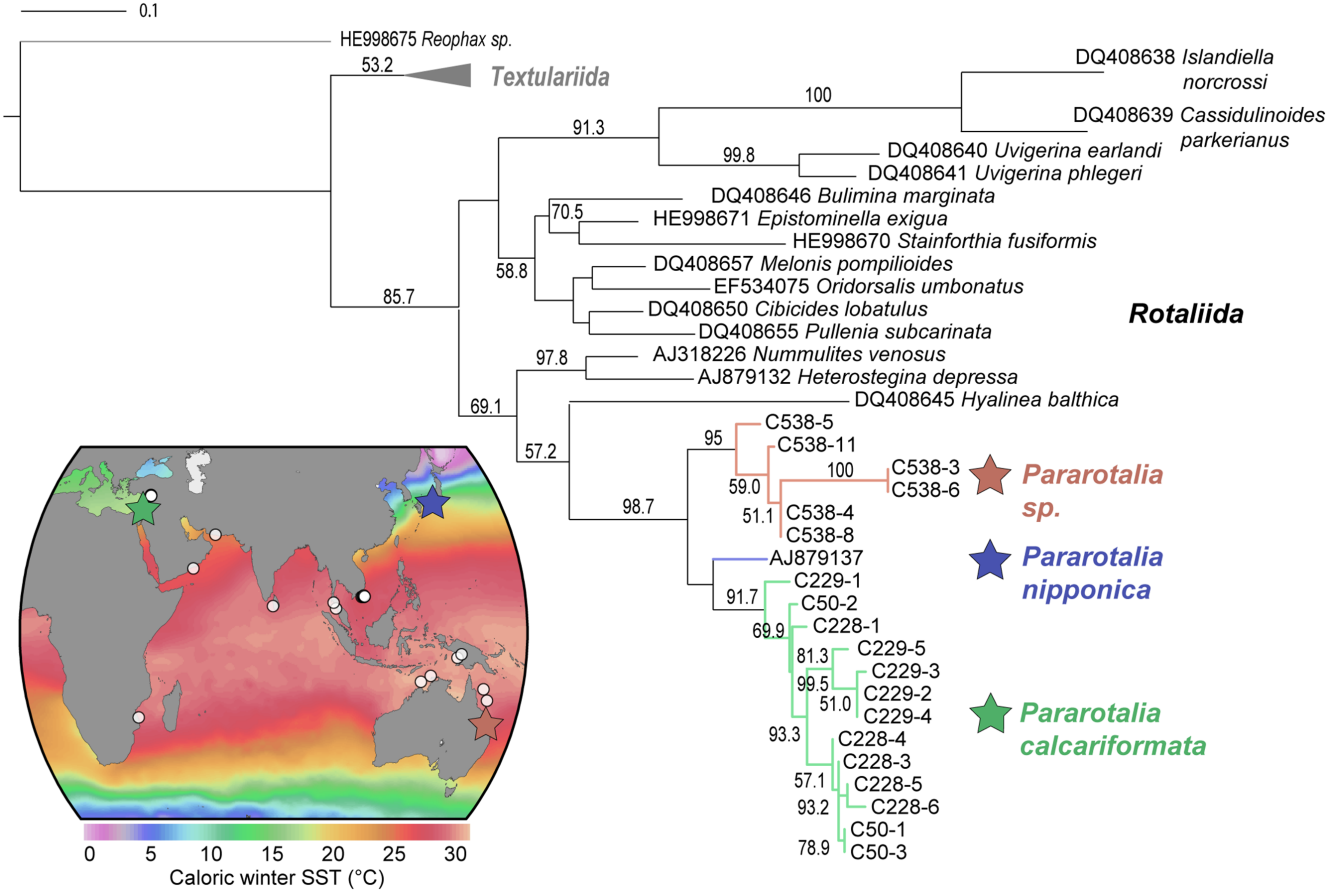
Phylogenetic tree (Maximum Likelihood, GTR+I+G) showing the evolutionary relationships of *Pararotalia* with other benthic foraminifera belonging to the order Globothalamea. Bootstrap scores (1000 replicates) higher than 50% are shown next to the branches. The tree is rooted on the genus *Reophax*. The occurrence of the genus in the Indo-Pacific is shown on the map against winter sea surface temperature (data extracted from [113]. White circles on the map represent the occurrences of *Pararotalia* reported in the literature (see S1 Table) and differently stars denote the locations of the sequenced specimens.

Having established its likely taxonomic identity and the range of morphologies represented in the Levantine populations (see S1 File), we next assessed the distribution of its potential parent populations. The species *P*. *calcariformata* was originally described by [52] from recent shallow habitats in Australia and Ceylon, and later reported throughout the Indopacific (see S1 Table). In addition, specimens closely resembling *P*. *calcariformata* have been identified from among SEM images available through the foraminifera.eu project [53] in faunas from Australian beaches, Malaysia, Oman and Iran (see S1 Table). These observations indicate that the species is a common element of tropical and subtropical assemblages throughout the Indopacific (Fig 2).

To confirm the Indopacific origin of the Levantine population using genetic inference, we have obtained SSU rDNA sequences from specimens collected from Israeli coast and specimens of a morphologically similar species of *Pararotalia* sp. from Australia and compared these with published sequences of *Pararotalia nipponica* originating from Japan. The relationship among these species was assessed by constructing a phylogeny rooted on agglutinated foraminifera and including representative sequences of all major calcareous clades, as presented in [54]. The resulting phylogenetic tree (Fig 2) has an almost identical topology to that inferred by [54], although the branch support is lower, most probably due to a shorter sequence length of the analyzed SSU fragment. All the obtained sequences of *Pararotalia* form a highly supported monophylum. The Levantine population sequences cluster within one clade and appear more closely related to sequences belonging to *Pararotalia nipponica*. This suggests that the Levantine population is derived from within the Indopacific radiation of the genus. The Australian *Pararotalia* sp. is similar to *P*. *calcariformata* in possessing spines, but these are much more regularly developed, such as in the species *P*. *stellata* (de Férussac, 1827). A clarification of the relationship among the three studied forms would require a comprehensive taxonomic revision of the group.

### Characterization of diatom endosymbionts and their photochemistry

The ecology of symbiont-bearing benthic foraminifera is closely tied to the function of their algal symbionts. Although the genus *Pararotalia* together with the genus *Neorotalia* is considered to belong to the informal group of larger benthic foraminifera (LBF), which are typically associated with algal symbionts [20], the presence and identity of the symbionts in *Pararotalia* has never been formally established. In addition, the Mediterranean *Pararotalia* is smaller (typically <400 μm) than other symbiont-bearing benthic foraminifera. One study reporting the occurrence of the invasive *Pararotalia* inside a thermally polluted site along the Israeli coast [31] noted a distinct coloration of its cytoplasm, which is often an indicator for the presence of algal symbionts. Therefore, prior to further physiological experiments, we investigated the presence and nature of symbionts in the studied Levantine population.

**Fig 3:**
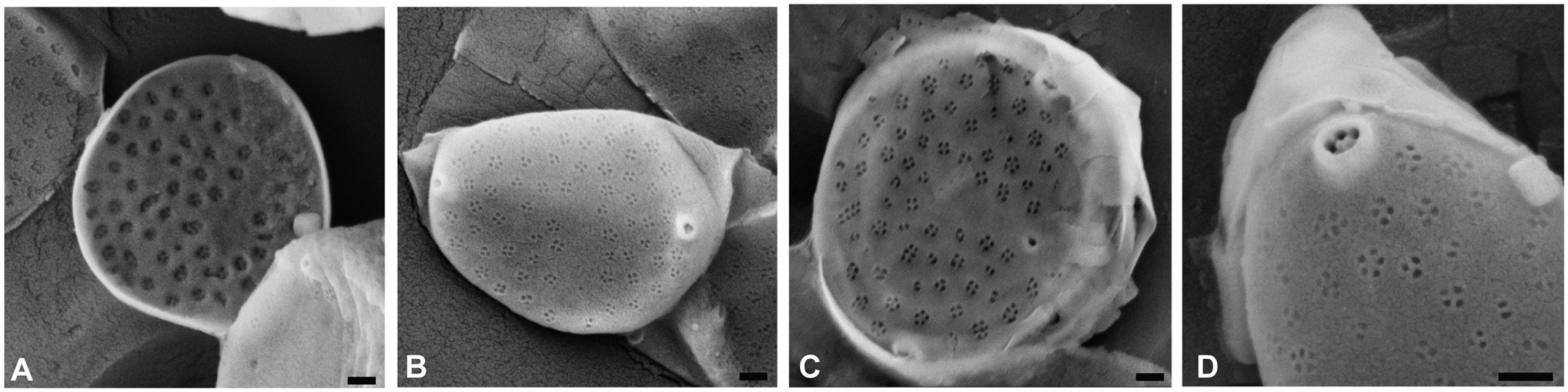
Scanning electron microscopy images of the endosymbiotic diatom *Minutocellus polymorphus* isolated from the foraminifer *Pararotalia calcariformata*. Samples of foraminifera were obtained from Nachscholim National Park, Israel. Symbionts were isolated in the laboratory and grown in sterile sea water media. Scale bars represent 200 nm.

In molecular phylogenies, *Pararotalia* appears to cluster with Calcarinidae and Nummulitidae [55] which are typically associated with diatom symbionts [56–58]. However, the diatom symbiosis in foraminifera is known to involve different and potentially multiple species [59], prompting us to use two methods to determine the identity of the symbionts in the investigated Levantine population. First, we isolated the symbionts by crushing the calcite shell, opening the protoplasm and growing the cellular content in axenic media. Endosymbiotic diatoms extracted and grown in this manner begin to produce frustules, allowing their morphological classification [60]. Symbiont cultures obtained from five specimens of *P*. *calcariformata* from the locality Nachsholim revealed, in three cases, the presence of multiple species of diatoms and in two cases only a single diatom species. In four cultures the diatom could be identified as *Minutocellus polymorphus* (Hargraves & Guillard) Hasle, Stosch & Syvertsen (Fig 3). In three cultures *Navicula* sp. was observed, whereas *Amphora bigibba* and *Amphora* sp. (with asymmetrical raphe) were only observed in one culture each.

The results of the symbiont culturing show that several species of diatoms can be identified within the same host. This is in line with previous work on related taxa [57]. So far, 20 diatom species or varieties have been isolated as potential symbionts of foraminifera [61]. Of these, *Nitzschia frustulum* var. *symbiotica* is the most commonly isolated diatom endosymbiont [62, 63]. Interestingly, we did not find this species in the cultures derived from *P*. *calcariformata*. In contrast, the most commonly identified potential symbiont *Minutocellus polymorphus* has never been observed in a benthic foraminifera host before [62]. This diatom is found free-living in the Mediterranean [64] and given its small size (up to 3 μm) it can plausibly act as a symbiont.

To confirm that this species was numerically abundant during life of the *P*. *calcariformata* holobiont, we amplified a SSU rDNA fragment of total DNA extractions from two specimens from the Nachsholim locality. The amplification was carried out using primers that were designed to anneal with a range of eukaryotic lineages but not the foraminifera host (see methods). In both specimens, the PCR (polymerase chain reaction) product yielded a single electrophoresis band, which could be directly sequenced, indicating the presence of a numerically dominant signal. The resulting sequences could be unambiguously identified as *Minutocellus* by comparison with the SILVA database [65]. Therefore, we conclude that the diatom endosymbiont consortium in the investigated specimens of *P*. *calcariformata* was likely dominated by *M*. *polymorphus*.

**Fig 4:**
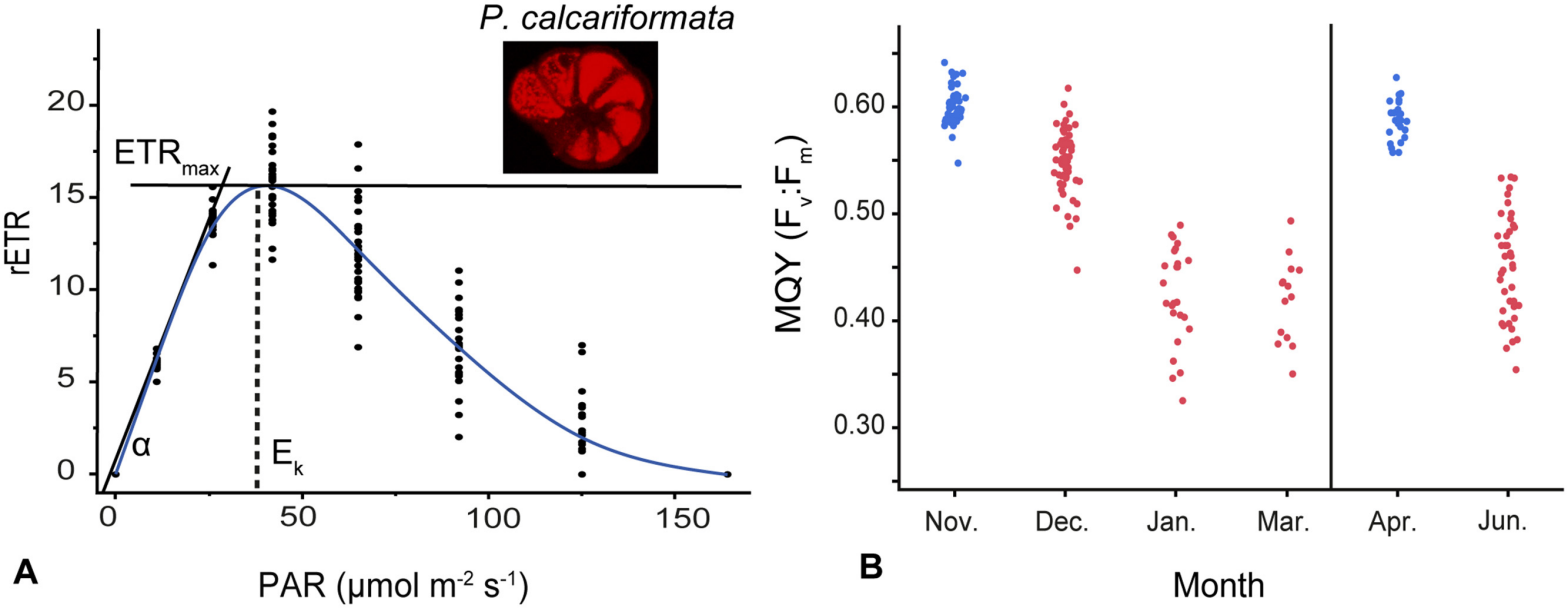
Photochemical activity in endosymbionts of the foraminifer *Pararotalia calcariformata*. A) Rapid light curve showing relative electron transport rates (rETR) under different levels of photosynthetically active radiation (PAR) on low-light adapted specimens (n = 30). The curve shows a cubic spline with a lambda of 0.05, α represents slope of the curve. ETR_max_ represents the maximum height of the curve and E_k_ the minimum saturating irradiance level. Small photograph shows a live specimen under epifluorescence microscopy containing photosynthetically active symbionts. B) Maximum quantum yield (F_v_:F_m_) measured on two populations collected in 11/2012 and 04/2013 (n = 14–48) and monitored in the laboratory for several month. Blue dots represent measurements made one week after collection. Red dots represent measurements of cultured specimens.

To characterize the photosynthetic functioning of the discovered diatom endosymbiont, we measured the photosynthetic activity of the symbionts inside the host by Pulse Amplitude Modulated Fluorometry (PAM) (Fig 4). To achieve this we conducted Rapid Light Curves (RLCs) after the protocol of [66], allowing us to assess the response of PS II (Photosystem II) to elevated light levels. The measurements on freshly collected specimens yielded an RLC (Fig 4A), most similar to intermediate-light adapted *Amphistegina* [67, 68]. We observed PSII photoinhibition (light adapted yields, Y(II) = 0) at 166 μmol photons m^-2^ s^-1^. The RLC of *P*. *calcariformata* thus reveals an unusual sensitivity to high irradiance among the foraminifera, which we attribute to the combination of the different nature of the symbiont and the mid-latitude setting of the locality. Alternatively, the observed lack of fluorescence at higher irradiance levels could be an artefact due to the behavior of the microalgae in the shell. On the other hand, the ETR_max_ (maximum height of the curve) and the E_k_ (minimum saturating irradiance level) of the curve fall exactly within the range of values determined for other diatom-bearing foraminifera [67, 68].

The observed sensitivity to in-situ light intensities in *P*. *calcariformata* are in line with earlier studies, who reported reduced F_v_:F_m_ and growth in *Calcarina* sp. cultured in high and mid-light treatments[68]. This can be explained by the fact that foraminifera are motile and live in microhabitats that allow them to shelter their shell from excessive light by hiding underneath stones, below the thalli of the *Jania rubens* macroalgae or other turf algae growing on bedrock. Because of the apparent light sensitivity of *P*. *calcariformata* the irradiance level in cultures and subsequent experiments was kept below 30 μmol photons m^-2^ s^-1^ PAR.

Next, PAM fluorometry was used to confirm the activity and persistence of the photosymbiosis among specimens collected during different seasons and in populations kept in culture for up to five months by measuring the dark adapted yield (maximum quantum yield; F_v_:F_m_). Field populations measured within one week of collection exhibit average F_v_:F_m_ of 0.60 in collections from both 11/2012 and 04/2013 (Fig 4), indicating a seasonally persistent fully functional photosymbiosis in the studied population. The values are comparable to those determined in previous work on other species of symbiont-bearing foraminifera [21, 67, 68]. After one month of culturing the F_v_:F_m_ of the population collected in 11/2012 decreased to an average of 0.55. After longer exposure to laboratory conditions, the average F_v_:F_m_ decreased further between 11/2012–03/2013 to 0.42 (70% of the initial value) and between 04/2013–06/2013 to 0.45 (76% of the initial value). In the foraminifera *Marginopora vertebralis*, F_v_:F_m_ between 0.15–0.38 were still considered to represent functional photosymbiosis of the dinoflagellates symbionts [69]. Thus, despite the reduced F_v_:F_m_, the *Pararotalia* specimens remained photosynthetically active in culture for several months, indicating that the symbiosis is of persistent nature.

The observed reduction of F_v_:F_m_ with time might be a sign of a reaction of the symbiont or the holobiont to the culturing conditions. In comparison to fluctuating light intensities and daily light peaks in their natural habitat [68] the cultured specimens were exposed to low and constant light levels. On the other hand, lower F_v_:F_m_ values could indicate nutrient stress in the cultures. It has been shown that low-light adapted diatoms have higher cellular iron needs to keep photosynthetic iron-based redox proteins functioning [70]. Since the cultures were based on artificial seawater without addition of nutrients, it is possible that the feeding of the foraminifera with microalgae was not sufficient to allow for optimal nutrition. In contrast to this hypothesis, nutrient-limited cultures of the diatom species *Thalassiosira pseudonana* exhibit a constant F_v_:F_m_ ratio of 0.65 under balanced growth conditions [71]. Therefore, it remains unclear whether the decreased F_v_:F_m_ indicates nutrient or light stress in the cultures, or whether it reflects the physiological state of the symbionts.

### Reproduction, growth and temperature sensitivity of asexual offspring

The extent of biological invasions and range expansions is limited by the ability of the species to establish a viable population at a new locality. Thus, next to the environmental tolerance of adult specimens, reproductive success of the expanding population is determined by the environmental suitability window of the reproduction event and of the survival and growth of juveniles, which can be a bottleneck for species survival under global change [72]. Foraminifera are known to reproduce through a complex system of sexual (gametogenesis) and asexual (multiple fission) cycles [73, 74]. The length of the life cycle and the timing of reproduction is often poorly constrained. Continuous in situ monitoring over the yearly cycle shows highest abundances of *P*. *calcariformata* in the >0.63 μm size fraction in spring following natural temperature rise [31], indicating that reproduction in this species in nature is likely to occur once a year in late spring.

Because we have observed asexual reproduction in populations in the laboratory cultures, we were able to characterize the ontogeny under controlled laboratory conditions. After collections in 11/2012 and 04/2013, reproduction occurred at least once in each collection after 4–5 weeks of culturing, involving only a small number of the cultured specimens. The number of offspring per mother individual was relatively small: after reproduction in 05/2013, we observed app. 400 living three-chambered offspring and app. 20 dead large parent individuals in the same culture. The offspring contained a minimum of three chambers when released from the parent and contained symbionts. The size and number of asexual offspring, as well as the presence of symbionts inherited from the parent are comparable to observations from laboratory experiments on many other species of symbiont-bearing foraminifera [74]. Thus, the Levantine invasive population of *P*. *calcariformata* is able to reproduce under temperature, salinity and light conditions simulating the ambient setting in the Levant in autumn and spring. However, development under culturing conditions can only be an estimate compared to development under in-situ conditions because environmental factors, e.g. tides and lunar cycles were not simulated [75, 76].

**Fig 5:**
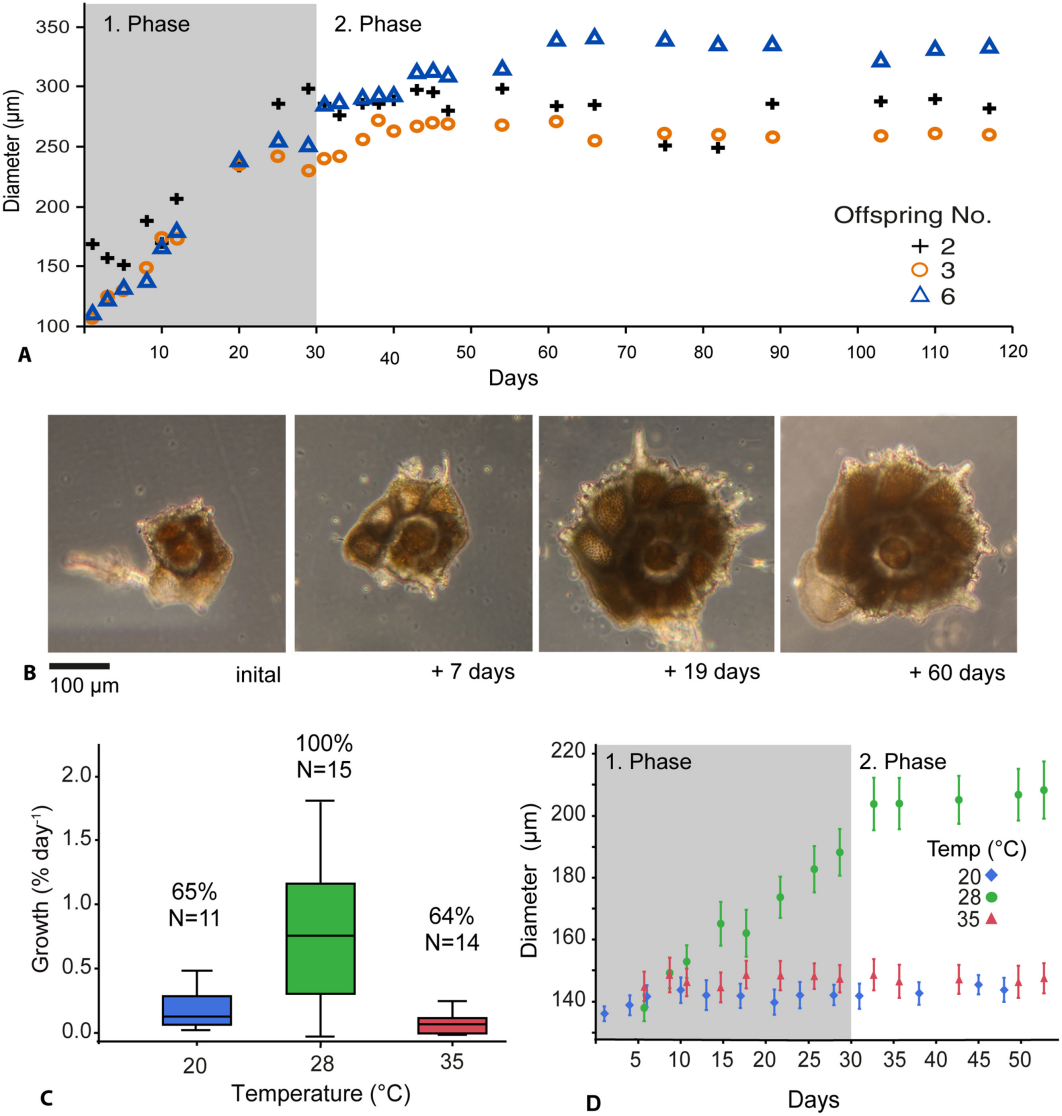
Ontogenetic shell development of the asexual offspring of the foraminifer *Pararotalia calcariformata*. A) Shell diameter (μm) of asexual offspring under culturing conditions showing rapid and slower growth phases. B) Images of consecutive ontogenetic stages of selected offspring Nr.2 from Fig 5A. C) Growth rates (% shell diameter increase day^-1^) of asexual offspring in temperature sensitivity experiment. Percentages indicate the number of individuals, which showed positive growth during the experiment. D) Mean diameter (μm) of all asexual offspring over the course of the temperature sensitivity experiment. Error bars represent 1 SE.

To characterize the growth of the asexual offspring, six juveniles from the first reproductive event were kept in culture under constant conditions for 117 days. Three of the six individuals grew and developed new chambers filled with green-brownish cytoplasm (Fig 5A and 5B). A twice weekly monitoring showed that the growth of the juveniles has two distinct phases. The first phase of rapid growth was characterized by a chamber number increase from three to 14–15 chambers lasting until ~ day 30 (Fig 5A). This rapid growth phase was followed by slower growth phase, were only 2–3 additional chambers were formed until termination of the experiment.

Following new collections in April 2013, we observed another reproductive event from which we sourced asexual offspring to investigate the growth and shell development under three temperatures 20°C, 28°C and 35°C. Asexual offspring were exposed to respective temperatures for 48 days. The 20°C and 28°C cultures were set to simulate the natural range of temperatures in the eastern Mediterranean between spring and autumn [31, 77], with the 20°C culture representing the conditions at the time of collection. The 35°C treatment was chosen to establish the upper limit of offspring growth.

The results indicated that offspring mean growth was 0.86% day^-1^ in the 28°C treatment (Fig 5C). Only in the 28°C treatment all observed specimens grew and the average growth curve of the population followed the same two-phase growth pattern as in the 2012 culture (Fig 5D). The offspring kept at 20°C and 35°C survived the experiment, as seen by healthy coloration of the cytoplasm and cytoplasmic movement. However, they exhibited an inhibition of growth, indicating that these levels represent lower and upper limits of growth (Fig 5D). This is in line with observations of benthic foraminifera where growth commenced only in individuals exposed to suitable environmental conditions [78]. The proportion of individuals which showed positive growth under experimental conditions was 100% under 28°C, and was reduced to 64–65% in the 20°C and 35°C treatment. We statistically tested the effect of temperature on the individuals exhibiting positive growth. Temperature had a significant effect on growth rate on asexual offspring (One-Way ANOVA, F = 24.60, df = 2, 34, p = 0.0001). The Tukey-Kramer post-hoc test revealed significant differences between the 28°C, the 20°C and 35°C treatments, but not between the latter two. Growth rates observed at 28°C in this study are comparable to those of sexually produced offspring of the benthic foraminifera *Planoglabratella opercularis* (0.4–0.8% growth per day between 15–20°C) inhabiting a similar coastal environment on coralline algae in Japan [79]. The observed inhibition of growth in the 35°C treatment, is consistent with an upper thermal limit for other species of symbiont-bearing foraminifera, where reduced growth, increased mortality and symbiont bleaching are observed at temperatures >31°C [21, 80].

In contrast, the lack of offspring growth at 20°C was unexpected, considering that this was the ambient temperature at the time of collection and the reproduction in the laboratory occurred at that temperature. Our results indicate that although reproduction may occur at 20°C, the offspring needs temperatures >20°C in the subsequent weeks in order to initiate the rapid growth phase. Thus, the observation of reproduction at 20°C may be consistent with the lower temperature limit for offspring growth between 20°C and 24°C, provided the reproduction is aligned with the onset of the spring warming. This is consistent with the observation of maximum abundance in June and July in the >0.63 μm size fraction [31]. If the elevated abundance reflects reproductive events and the natural population follows the same growth pattern as seen in laboratory, then the reproductive season in nature must have occurred at least one month before the observed abundance maximum, i.e. in May or June.

If temperatures higher than 20°C are required for offspring growth in *P*. *calcariformata*, then the seasonal time window for reproduction in this species is longer in the Levant than in other areas of the Mediterranean [81]. It is possible that reproductive processes in *P*. *calcariformata* are incompatible with a narrower suitability window for reproduction shifted towards the summer in other areas of the Mediterranean. At present, between the Levant (e.g., Haifa) and the Ionian Sea (e.g. San Stefano, Corfu) the 20°C sea surface temperature threshold is shifted by three weeks from early May to early June and the total length of the >20°C thermal window is shorter by two months [82]. This mechanism would provide a possible explanation for the restricted Levantine occurrence of the invasive species, as well as for its apparently recent invasion. The Levantine basin is known to have already experienced significant winter warming in the past decades and is predicted to increase its yearly mean SST by 0.5–2.3°C by the end of the century [25, 81].

### Modelling the present and future distribution of the species

If the limited distribution of *P*. *calcariformata* and its recent appearance in the Levant reflect low tolerance to temperatures at 20°C or below, then it should be possible to express all the known occurrences of the species as a function of the local yearly temperature cycle. To explore this hypothesis we combined published occurrence records of the species with own sampling in the Mediterranean (see S1 Table) and used the data to calibrate a species distribution model linked to environmental variables at the observed sites. The variables we considered include solar irradiance and turbidity, which are relevant to the symbiont photosynthesis and yearly minimum temperature, representing potential limiting factors for the holobiont. The values that were used to calibrate the model represent multi-year averages for a time period after the first record of the invasive species (1997–2009 for irradiance and 2002–2009 for temperature and turbidity), as used in previous studies [83].

**Fig 6:**
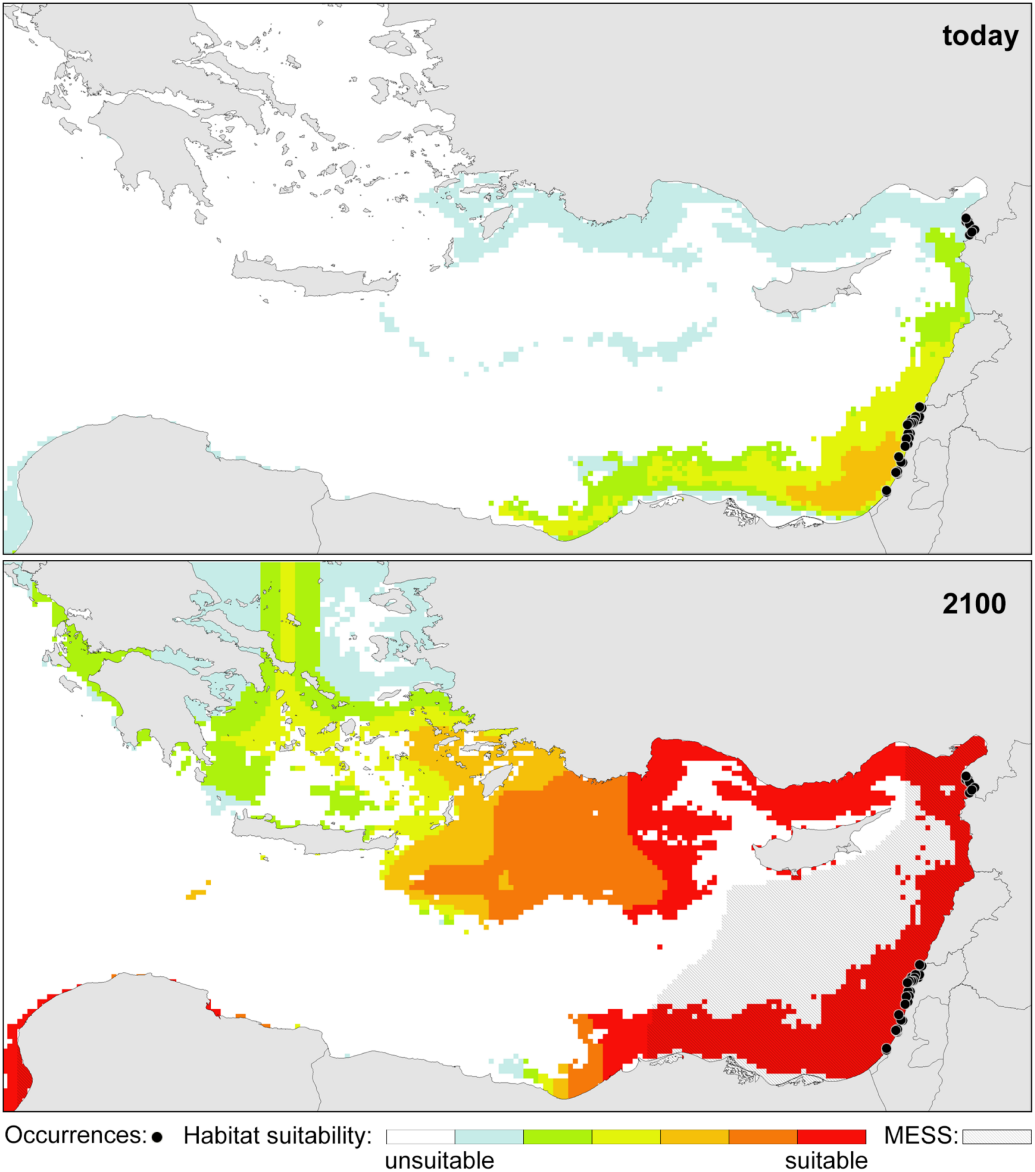
Occurrence records of the foraminifer *Pararotalia calcariformata* in the Mediterranean for present day and projected future conditions expected by 2100. Occurrence records are overlain with habitat suitability values from a species distribution model based on yearly minimum temperature, mean light attenuation and mean photosynthetically active radiation for the present day and under projected future conditions (intermediate global change scenario AB1). The results of the multivariate similarity surface analysis (MESS) are indicated by grey stripes in the future scenario model.

The resulting species distribution model (SDM) (Fig 6) indicates the highest habitat suitability in the Levant, along the coast of Israel and Lebanon. Moderately suitable habitat, representing “typical” conditions for a species, continues along the coast of Syria to southernmost Turkey, with its front corresponding exactly with the localities, where the invasive species has been most recently reported by [32]. The model also indicates the existence of suitable habitats along the Egyptian shelf, where the species has not been discovered so far [84, 85]. However, we note that the most suitable habitat in this region is inferred to exist offshore, being reflected already in the three variables driving the model. Anthropogenic contribution of nutrients from agriculture and sewage has replaced the nutrient stimulating effect of the Nile discharge after closure of the Aswan Dam in 1965 [86, 87], keeping conditions still sub-optimal for nutrient-sensitive organisms. Along the Levantine coast, *P*. *calcariformata* appears to preferentially inhabit the shallowest subtidal environment, above the depth of 20m, indicating that this species needs well-lit, oligotrophic conditions. We note that similar to other benthic symbiont-bearing foraminifera, *P*. *calcariformata* might therefore be a good indicator species for the FORAM Index as a proxy for ecosystem health in the Mediterranean, as shown for *Amphistegina lobifera* in the Aegean Sea [88]. In the studied localities, *P*. *calcariformata* lives in association with turf or calcareous algae that inhabit rocky substrate in the sub tidal and intertidal zone on abrasion platforms and beach rocks. As the Nile delta provides habitat lacking these conditions and because of the elevated nutrient load [87], it is not surprising that it may function as a natural barrier to a south-easterly coastal dispersal of the species.

Other species of *Pararotalia* have been reported to disperse with surface ocean currents attached to gastropod larvae [46] or fish [17]. Whether or not attached to gastropod larvae, macroalgae or transported as propagules [15, 78], the dispersal of *P*. *calcariformata* would lead to passive northward transport along the Levantine coast following the persistent surface currents in the Levant [89, 90]. These currents move directly along the Israeli shelf and could facilitate the transport of plant material or larvae carrying attached *Pararotalia* towards the northern Levant, as it has been suggested for other benthic foraminifera [26]. This would mean that the main direction of current transport of the Levantine *Pararotalia* is further to the north, along the coast of Turkey and into the Aegean Sea. Here, the SDM indicates poor habitat suitability, due to colder temperatures (Fig 6). Indeed, the species does not appear to have invaded the Aegean Sea yet [88, 91]. A recent detailed investigation of foraminifera diversity in the Saros Bay, a region with abundant suitable habitats for *P*. *calcariformata*, has not identified this species among the 115 species recorded [91].

To investigate whether the species range given by the SDM derived from its present-day distribution is likely to expand further, we have evaluated habitat suitability under a realistic (intermediate scenario A1B) global change projection for the year 2100 [92]. Predicted habitat suitability increases drastically along the coast of Turkey, suggesting a high probability of invasion in this region within the next decades. In addition, the results imply a large potential for an ongoing expansion of the species into the Aegean Sea and the Greek and Libyan coasts of the Ionian Sea.

## Conclusions

A taxonomic revision of the most recently discovered invasive species of symbiont-bearing foraminifer of the genus *Pararotalia* in the eastern Mediterranean identifies the invader as *P*. *calcariformata* McCulloch 1977. Based on phylogenetic and taxonomic evidence, the invasive population appears to have originated from within the Pacific radiation of the genus. Pulse amplitude modulated fluorometry measurements indicate that the species is engaged in permanent symbiosis with photosynthesizing microalgae. Combined culturing and genotyping approach allowed us to identify these algae as a consortium of small diatoms, including the newly described symbiont *Minutocellus polymorphus*.

*Pararotalia calcariformata* has been observed to asexually reproduce and grow in manipulative experiments, revealing an initial 30-day period of rapid growth followed by a slower growth phase. Reproduction occurred at 20°C, but normal offspring growth was only observed at 24°C and 28°C, indicating that a successful establishment of populations of the species may be limited by the length of the thermally defined reproductive window in their habitat. To test this hypothesis we derived a SDM based on present day occurrence of the species and show that together with turbidity and irradiance, yearly minimum temperature alone is sufficient to reproduce the observed species range with remarkable fidelity. This model indicates that the species is likely to continue expanding northwards and westwards under realistic global change scenarios and is likely to reach the Ionian Sea by 2100.

Collectively, the evidence indicates that the symbiont-bearing foraminifera *P*. *calcariformata* is likely a Lessepsian migrant whose invasion into the Mediterranean has been facilitated by the recent warming in the Levant [81]. In this way, the case of *P*. *calcariformata* adds to mounting evidence for ongoing and dramatic changes in the structure of eastern Mediterranean ecosystems [6, 11]. Its invasion reflects multiple aspects of human-mediated dispersal of marine species—environmental change due to global warming and removal of physical barriers, such as the opening or widening of the Suez Canal. The observed migration pattern indicates that like many other species which are not limited by dispersal, the invasion rate of *P*. *calcariformata* is high, resulting in an ongoing range expansion [32] observable on sub-decadal time scale.

## Material and Methods

### Sample collection and maintenance of cultures

Living specimens of *Pararotalia calcariformata* were collected during four field campaigns on 23/10/2012, 01/11/2012, 08/04/2013 and 14/04/2013 at Nachsholim and at Hadera, Israel at depths between 0.5–7 m (see S1 Table). Samples were collected by sampling macroalgae substrate (most commonly the coralline algae *Jania rubens*) by snorkeling or SCUBA diving. Living specimens of *P*. *calcariformata* and algae material were collected under permission number 2013/39805 issued by the Israel Nature and National Park Authority. Samples were transported in large plastic bottles filled with algae and sediment to the laboratory, where the algae and sediment were rinsed with sea water and specimens were picked from the concentrated sediment and put to a maximum number of 50 specimens in screw capped plastic jars (volume 100 mL). The jars were shipped inside an insolation container to Germany (express shipment time 48 h). The specimens were cultured in plastic containers at in-situ sea water temperatures (23–24°C in December, 20–21°C in April) under a diurnal 12 h/12 h light cycle, salinity 38.5–40 ‰ and irradiance of <30 μmol photons m^-2^ s^-2^. The seawater was prepared from artificial sea salt (Tropic Marin Sea Salt, Germany) every two weeks. Approximately 50% seawater was exchanged weekly and the cultures were fed with *Nannochloropsis* food mixture every 6–8 weeks. Food mixture contained algae concentrate (12 x 10^9^ cells/mL, BlueBioTech GmbH, Germany), which was diluted with artificial seawater (30 μL *Nannochloropsis* concentrate: 200 mL artificial seawater) and autoclaved. The sample of *Pararotalia* sp. from Pelican Island, Australia was collected on the 05/05/2014, sent by express shipment to Bremen and cultured under the above conditions and salinity of 35–36 ‰ prior to DNA analysis.

### Taxonomic identification and habitat

The morphology of *P*. *calcariformata* from the Israeli coast has been documented using SEM (Scanning electron microscopy) and light microscopy with a digital camera (Leica, DFC290HD) (Fig 1). A detailed taxonomic description of *P*. *calcariformata* is provided in S1 File, including SEM images showing adult and juvenile stages (see S1 File, Plate A, B). All samples for taxonomic identification originated from Nachsholim National Park. Occurrence records in the Mediterranean were obtained by literature search and our own sampling. We also carried out a literature search for *Pararotalia* morphological forms named as or resembling *P*. *calcariformata* in the Indopacific (S1 Table, color map Fig 2).

### Symbiont culturing and preparation for SEM microscopy

We have isolated and cultured the symbionts of five specimens of *P*. *calcariformata* collected in Nachsholim (see S1 Table) on 23/10/2013. The specimens were taken from cultures collected on 8/11/2013 and the isolates were grown in standard culture media (Guillard’s (f/2) Marine Water Enrichment Solution, Sigma Aldrich) [56, 60]. Cultures were terminated after 4 weeks of growth, oxidized by H_2_O_2_, filtered onto Nucleopore Track Etch Polycarbonate filters (Whatman) that were cut to fit the size of the metallic stubs used for examination in the SEM.

### PAM Fluorometry

To prove that *P*. *calcariformata* establishes a permanent photosymbiosis with algae we characterized its photosynthetic activity by Pulse Amplitude Modulated (PAM) fluorometry. For measurements of dark adapted yield (maximum quantum yield, MQY, F_v_:F_m_) of Photosystem (PS) II, IMAGING-PAM M-Series fluorometer (IPAM, WALZ GmbH, Germany) was used. It was equipped with MAXI-Head, 1/2” CCD camera and zoom objective (F1.0/f = 8–48 mm). Specimens were transferred in Petri dishes containing fresh seawater and dark-adapted 10–15 minutes before measuring F_v_:F_m_. We elevated the Petri dishes closer to the zoom objective on a 1.5 cm high stand and used the Leaf Holder IMAG-MIN/BK to allow best possible imaging for all specimens (size 0.3–0.4 μm). Other procedure followed the protocols previously published [21, 80, 93, 94].

Using the IPAM we also measured rapid light curves (RLC) to access photosynthetic activity in the symbionts in *P*. *calcariformata* [66] under different light intensities. After each light intensity step, the effective quantum efficiency (Y(II)) of the symbionts is automatically measured by the Imaging Win Software (WALZ, Germany). Dark adaptation was chosen to be for 10 s [66], and followed by several increasing light intensities for 10 s each. The irradiance steps emitted by the LEDs of the IPAM instrument were calibrated using a hand-held PAR Light Meter (Apogee, USA) and were as follows: 0, 11, 26, 42, 65, 92, 125, 164, 213, 264, 313, 385, 450, 534, 604, 682, 780 μmol photons m^-2^ s^-1^. For every irradiance level, the relative electron transport rate (rETR = E × Y(II)) was calculated and rETR versus Apogee PAR light intensity (E) were plotted (Fig 4A). Several photosynthetic parameters were drawn in the curve for illustration such as ETR_max_ (maximum height of the curve), the E_k_ (minimum saturating irradiance level) and the slope of the curve (α), which aid in determinging the photosynthetic activity level. However, as we do not intent to compare the response between different light levels or species, we simply estimated them based on a standard curve (cubic spline with a default lambda of 0.05) fitted by Jmp 11 [95]. Measurements on 17/04/2013for the RLC were conducted on 30 randomly selected specimens three days after sampling, pre-adapted to light levels <30 μmol photons m^-2^ s^-1^ levels.

### Reproduction and offspring experiments

To test whether the asexual offspring can be cultivated under laboratory conditions we randomly choose six juveniles that were found in the culture in December 2012 and monitored their development for 117 days (12/2012–04/2013). To this end we used a 6-well plate filled with artificial seawater (~15 ml volume per well), closed with a lid and placed inside an incubator illuminated at 10–15 μmol photons m^-2^ s^-1^ (Leec Plant Growth Cabinet, Model PL2, 150 litre, UK), as tested in previous work [21]. *Nannochloropsis* food mixture (see general culturing) was added to each well on11/12/12, 22/12/13, 11/01/13 and 08/03/13. The juveniles were transferred to a new plate before each feeding to ensure the same conditions in each well. Water was exchanged by 50% twice weekly and the specimens were photographed and measured. Salinity was monitored with a handheld salinity and temperature meter (WTW and Oregon Scientific, USA) before each water exchange. Salinity stayed between 38.9–39.3 ‰ during the culturing period, simulating natural conditions. Temperature of the incubator was adjusted to natural winter conditions in the Levant during the culturing period (for 12/12 from 23–24°C followed by a slightly colder period from 01/13–03/13 (20–22°C).

Following the initial culturing of a small number of asexual offspring, we tested the development of the asexual offspring under different temperatures (20, 28, and 35°C) in a shorter and replicated design using 54 individuals. These were randomly selected from ~400 juveniles from the May 2013 reproduction event and cultured for 48 days (05/2013–07/2013). Three thermostatic cabinets (Pol-Eko-Aparatura, Model ST2+/ST2+, Poland) were used for the setup containing three 6-well plates each with 18 specimens exposed to the same illumination level in the incubator. All plates received the same light conditions (12-hour cycle, 25–30 μmol photons m^-2^ s^-1^) throughout the experiment. To ensure that growth was not influenced by the location of the plate inside the incubator, the order of the plates was changed randomly when the water was exchanged three times weekly. Light levels were chosen to be higher than in the previous culturing work because rapid light curves (RLC) from 04/2013 indicated better performance of the photochemistry of the symbionts (Fig 4A) under slightly elevated conditions. *Nannochloropsis* food mixture (see general culturing) was added to each well at the onset of the experiment at 18/05/13 and at 19/06/13. A more frequent feeding was not necessary as enough food was still visible in the plates. Water exchange was performed three times weekly and manual monitoring of salinity and temperature was done in the same way as described before. At the onset of the experiment temperatures in incubators were slowly ramped up to prevent acute temperature stress. The 28°C and 35°C incubator were automatically ramped up to final temperature over a period of 12 h (2°C every 2–3 h). Manual temperature measurements (n = 57) conducted inside the plates showed that actual temperatures in the incubators varied minimally 20.3°C (SD 0.1°C), 27.7°C (SD 0.2°C) and 34.7°C (SD 0.3°C). During the first 72 h of the experiment salinity peaked by +2–4 ‰ above the target levels at 38.5–40‰, which otherwise remained constant inside the wells throughout the experiment (Mean 39.7 ‰, SD 1.1). This short time span of elevated salinity did not cause mortality or bleaching, as the species has been shown to inhabit high saline areas [96] and is generally adapted to very saline water of the Levant [77]. The pH of the artificial ocean water was monitored weekly with a handheld meter (WTW, Germany) and stayed between 8.1–8.2 units.

### Growth measurements

Twice weekly during culturing experiments specimens were photographed inside each well (Fig 5B) with an inverse microscrope (Zeiss PrimoVert) and images were taken at the resolution of 5184 x 3456 pixel (Canon SLR camera). Largest diameters were measured starting at the last build chamber diagonally across the shell, using image analyzing software (ImageJ). Growth rates (% diameter increase day-1) were calculated using the formula given in [21] adapted from [97].

At the end of the temperature sensitivity experiment 46 of initially 54 specimens were retrieved for measurements (1/18 went missing in 20°C, 3/18 in 28°C, 4/18 in 35°). Although, the shell diameter increased in most specimens throughout the experiment (=positive growth, indicated in % in Fig 5A), we observe apparent negative growth rates in 35% of the specimens in the 20°C treatment (6/17 specimens) and 36% in the 35°C treatment (5/14 specimens). The negative growth reflects measurement uncertainty due to uneven orientation and attached debris, and is interpreted to indicate lack of growth. To exclude the possibility that the observed positive growth rate also represent measurement uncertainty, the total number of chambers was counted for specimens where the shell was best exposed. In both the 20°C and the 35°C treatments, we could identify specimens that added at least one chamber during the experiment. One-way Analysis of Variance (ANOVA) was used to test the effect of temperature on growth rates of asexual offspring. Statistics were performed on all individuals exhibiting positive growth rates (Fig 5A). Growth data was 3^rd^ root transformed and residual and normality plots indicated that equal group variance was not violated. ANOVA was performed using Jmp 11 [95].

### DNA extraction, amplification, cloning and sequencing

Three specimens of *P*. *calcariformata* collected from Hadera Ridge, Israel (C50) and in Nachsholim, Israel (C228 and C229) on 23/10/2013 and one specimen of *Pararotalia* sp. collected in Pelican Island, Australia (C538) on 05/2014 were isolated from cultures into 50 μl of GITC* on 07/11/2013 and 26/05/2014. Because of the thickness of the shell, the specimens were crushed with a metal rod prior to DNA extraction. A fragment of ~1000 bp located at the 3’ end of the SSU rDNA of the foraminifer was amplified using the primer couple S14F1 (5'-AAGGGCACCACAAGAACGC-3’)– 1528R (5'-TGATCCTTCTGCAGGTTCACCTAC-3’) by Polymerase chain reaction (PCR) [98, 99] using the GoTag (Promega, USA) or PHUSION (Thermo Scientific, USA) polymerase. The PCR products were purified using the QIAquick PCR purification kit (Qiagen, Netherlands) and cloned with the Zero Blunt TOPO PCR Cloning Kit (Invitrogen, USA) with TOP10 chemically competent cells following manufacturer’s instructions. Three to six clones were sequenced per individual by an external provider (LGC Genomics, Berlin). The obtained sequences were deposited on NCBI under the accession number KP939152 to KP939156 and KP939158 to KP939171.

A fragment of ~400 bp of the 3’ end of the SSU rDNA of foraminifers’ symbionts was obtained from aliquots of the same DNA extractions using the GoTaq polymerase (Promega, USA) with the symbionts specific forward primer SymSF1 (5'-GGTTAATTCCGTTAACGAACGAGA-3’) coupled with the universal reversed primer 1528R (5'-TGATCCTTCTGCAGGTTCACCTAC-3’) for the specimen C228 and C229. No multiple bands have been observed after migration of the PCR product on agarose gel. The PCR products were purified using the QIAquick PCR purification kit (Qiagen, Netherlands) and directly sequenced. The sequence chromatograms were carefully checked and no sign of multiple signals was detected. The obtained sequences were deposited on NCBI under the accession number KP939151and KP939157.

### Sequence analysis

The obtained 19 sequences of *P*. *calcariformata* were analysed together with 24 sequences of benthic foraminifera belonging to the lineage of *Globothalamea* [54] downloaded from GenBank (see S2 File). The sequences were automatically aligned with MAFFT v.7 [100] with default options. Only the fragment covered by the obtained sequences of *Pararotalia* was retained for further analyses (see S2 File). The model of evolution (GTR+I+G) was selected using jModeltest v. 2.1.4 [101] under Akaike Information Criterion (AIC). Using this model of evolution, the most likely tree topology was inferred from the alignment using a Maximum Likelihood Approach implemented in PhyML 3.0 software [102], using NNI+SPR tree improvement and non-parametric bootstrapping (1000 pseudo replicates). The resulting tree was visualized with iTOL v 2.1 (Fig 2) [103]. The two symbiont sequences were compared to the SILVA database [65] on the 21/08/2014 in order to determine their most probable taxonomic assignation. The SINA 1.2.11 alignment tool [104] has been used with default options.

### Computation of the habitat model

Occurrence records of *P*. *calcariformata* in the Mediterranean were obtained by literature search and combined with new observations during this study (see S1 Table). For the calibration of the species distribution model (SDM), occurrences were converted to presence records on a grid used by the modeling software. Environmental data for these grid cells were obtained from the BIO-Oracle database, which provides oceanographic variables with a grid-cell resolution of 5 arc minutes [105]. BIO-Oracle also includes gridded data from climate model projections that are based on SRES climate-change scenarios [92] and for our model we used the intermediate scenario A1B for the a projection to year 2100. We based the SDM for *P*. *calcariformata* mainly on temperature (annual minimum SST) and added annual mean diffuse attenuation (mean DA) and annual mean photosynthetically available radiation (mean PAR). The latter variables provided the possibility to incorporate the effects of terrestrial and trophic influences, as well as solar radiation on the potential distribution. These variables have been proven useful in previous modeling calibrations from other symbiont-bearing foraminifera [106]. The resulting SDM was refined in a two-step clipping process in order to avoid a biased relation between the variables, an approach that has been successfully used in other models on foraminiferal distributions [107]. First, we used only minimum SST, which was subsequently projected on the future climate scenario. Second, we built a model on mean DA and mean PAR. The final SDM for both current and future conditions (Fig 6) thus comprises a climate-model based on temperature (including the projection on the A1B scenario), which was overlain and clipped by a habitat-model based on the other variables. The editing of the climate model was performed with the software DIVA-GIS.

We used Maxent 3.3.3k [108] to model the potential distribution of *P*. *calcariformata* in the eastern Mediterranean and to project it onto future climate conditions. The program uses a grid-based machine-learning algorithm following the principles of maximum entropy [109]. In the course of the modeling process, Maxent begins with a uniform distribution and successively fits it to the data (occurrence records and environmental variables). For an overview on the operating mode of Maxent and the interpretation of its output see [110]. Note that Maxent does not predict the actual distribution of the taxon, but rather the relative suitability of the habitat, which is interpreted as the potential distribution of the taxon under study. A total of 10,000 random background points were automatically selected by Maxent within the eastern Mediterranean. The logistic output format with suitability values ranging from 0 (unsuitable) to 1 (optimal) was used [111], where the probability of presence at sites with "typical" conditions is set to 0.5 by default [110]. The modeling process was performed with 50 replicates and the average predictions across all replicates were used for further processing. The continuous probability surfaces of the SDMs were subsequently converted into presence/absence maps using the “Equal training sensitivity and specificity logistic threshold” as recommended by [112], which has also been used in previous foraminiferal models [106].

Projecting a model on future climate scenarios may result in an extrapolation or “clamping” of the probability values [108] especially in regions where the environmental predictors are outside the training range, which could lead to an over- or underfitting of the model. In Maxent, a multivariate similarity surface (MESS) analysis is implemented, which shows how similar predictor variables within future climate scenarios are seen during model training [110]. We added the result of the MESS analysis to our future model, highlighting areas of possible extrapolation of the model due to minimum temperature values of the future scenario being outside the training range.

## Supporting Information

S1 File Taxonomic species description of the foraminifera *Pararotalia calcariformata*. This file includes a documentation of shell morphology of late ontogenetic stages and their specific features (Plate A) and juvenile from field collections (early ontogenetic stages, Plate B) in Nachscholim National Park, Israel.
(DOCX)

S2 File DNA sequence alignment of *Pararotalia calcariformata* and other benthic foraminiferal species. This data was used for the phylogenetic inference, as shown in Fig 2.
(TXT)

S1 Table Occurrence records of the foraminifera *Pararotalia calcariformata*. Occurrence records in the Mediterranean have been used to develop the species distribution model (SDM) shown in Fig 6. Indo-pacific occurrence records of *Pararotalia calcariformata* are shown from line 97–116, which are shown on the world map in Fig 2.
(XLSX)

## References

[1] Vitousek PM, Mooney HA, Lubchenco J, Melillo JM. Human domination of earth's ecosystems. Science. 1997;277(5325):494–9.

[2] Sorte CJB, Williams SL, Carlton JT. Marine range shifts and species introductions: comparative spread rates and community impacts. Glob Ecol Biogeogr. 2010;19(3):303–16. 10.1111/j.1466-8238.2009.00519.x WOS:000276490400002.

[3] Chen IC, Hill JK, Ohlemuller R, Roy DB, Thomas CD. Rapid range shifts of species associated with high levels of climate warming. Science. 2011;333(6045):1024–6. 10.1126/science.1206432 WOS:000294000400057. 21852500

[4] Carlton TC. The scale and ecological consequences of biological invasions in the World's oceans In: Sandlund OT, Schei JP, Aslaug V, editors. Invasive Species and Biodiversity Management, Based on a selection of papers presented at the Norway/UN Conference on Alien Species, Trondheim, Norway. Dordrecht, Netherlands: Kluwer Academic Publishers 1999.

[5] Parmesan C. Ecological and evolutionary responses to recent climate change. Annu Rev Ecol Evol Syst. Annual Review of Ecology Evolution and Systematics. 372006 p. 637–69.

[6] Hiddink JG, Lasram FB, Cantrill J, Davies AJ. Keeping pace with climate change: what can we learn from the spread of Lessepsian migrants? Global Change Biology. 2012;18(7):2161–72. 10.1111/j.1365-2486.2012.02698.x WOS:000304820300007.

[7] Belanger CL, Jablonski D, Roy K, Berke SK, Krug AZ, Valentine JW. Global environmental predictors of benthic marine biogeographic structure. Proc Natl Acad Sci U S A. 2012;109(35):14046–51. 10.1073/pnas.1212381109 WOS:000308565300040. 22904189 PMC3435205

[8] Rilov G, Galil B. Marine bioinvasions in the Mediterranean Sea—History, Distribution and Ecology In: Rilov G, Crooks J, editors. Biological Invasions in Marine Ecosystems. Ecological Studies. 204: Springer Berlin Heidelberg; 2009 p. 549–75.

[9] Zenetos A, Gofas S, Morri C, Rosso A, Violanti D, Raso JEG, et al Alien species in the Mediterranean Sea by 2012. A contribution to the application of European Union's Marine Strategy Framework Directive (MSFD). Part 2. Introduction trends and pathways. Mediterranean Marine Science. 2012;13(2):328–52. WOS:000315934300019.

[10] Macias D, Garcia-Gorriz E, Stips A. Understanding the causes of recent warming of Mediterranean waters. How much could be attributed to climate change? Plos One. 2013;8(11). 10.1371/journal.pone.0081591 WOS:000327652100087. PMC3842300 24312322

[11] Edelist D, Rilov G, Golani D, Carlton JT, Spanier E. Restructuring the Sea: profound shifts in the world's most invaded marine ecosystem. Divers Distrib. 2013;19(1):69–77. 10.1111/ddi.12002 WOS:000312534700007.

[12] Langer MR, Weinmann AE, Lötters S, Rödder D. "Strangers" in paradise: modeling the biogeographic range expansion of the foraminifera Amphistegina in the Mediterranean Sea. J Foraminifer Res. 2012;42(3):234–44.

[13] Merkado G, Holzmann M, Apotheloz-Perret-Gentil L, Pawlowski J, Abdu U, Almogi-Labin A, et al Molecular evidence for Lessepsian invasion of Soritids (larger symbiont bearing benthic foraminifera). Plos One. 2013;8(10). 10.1371/journal.pone.0077725 WOS:000326270700052. PMC3812231 24204936

[14] Alve E. Colonization of new habitats by benthic foraminifera: a review. Earth-Sci Rev. 1999;46(1–4):167–85. 10.1016/S0012-8252(99)00016-1

[15] Alve E, Goldstein ST. Propagule transport as a key method of dispersal in benthic foraminifera (Protista). Limnol Oceanogr. 2003;48(6):2163–70. WOS:000186772800009.

[16] Caruso A, Cosentino C. The first colonization of the genus Amphistegina and other exotic benthic foraminifera of the Pelagian Islands and south-eastern Sicily (central Mediterranean Sea). Mar Micropaleontol. 2014;111(0):38–52.

[17] Debenay J-P, Sigura A, Justine J-L. Foraminifera in the diet of coral reef fish from the lagoon of New Caledonia: Predation, digestion, dispersion. Rev Micropaleontol. 2011;54(2):87–103.

[18] McGann M, Sloan D, Cohen AN. Invasion by a Japanese marine microorganism in western North America. Hydrobiologia. 2000;421:25–30. 10.1023/a:1003808517945 WOS:000086541800002.

[19] Rahel FJ, Olden JD. Assessing the effects of climate change on aquatic invasive species. Conserv Biol. 2008;22(3):521–33. 10.1111/j.1523-1739.2008.00950.x 18577081

[20] Langer MR, Hottinger L. Biogeography of selected "larger" foraminifera. Micropaleontology. 2000;46:105–26.

[21] Schmidt C, Heinz P, Kucera M, Uthicke S. Temperature-induced stress leads to bleaching in larger benthic foraminifera hosting endosymbiotic diatoms. Limnol Oceanogr. 2011;56(5):1587–602. 10.4319/lo.2011.56.5.1587 WOS:000294603500004.

[22] Hallock P. Algal symbiosis: a mathematical analysis. Mar Biol. 1981;62(4):249–55. 10.1007/BF00397691

[23] Lee JJ, Hallock P. Algal symbiosis as the driving force in the evolution of larger foraminifera. Ann N Y Acad Sci. 1987;503(Endocytobiol.):330–47. 10.1111/j.1749-6632.1987.tb40619.x

[24] de Nooijer LJ, Toyofuku T, Kitazato H. Foraminifera promote calcification by elevating their intracellular pH. Proceedings of the National Academy of Sciences. 2009;106(36):15374–8. 10.1073/pnas.0904306106 PMC2741258 19706891

[25] Sisma-Ventura G, Yam R, Shemesh A. Recent unprecedented warming and oligotrophy of the eastern Mediterranean Sea within the last millennium. Geophys Res Lett. 2014;41(14):2014GL060393 10.1002/2014GL060393

[26] Langer MR. Foraminifera from the Mediterranean and the Red Sea Aqaba-Eilat, the Improbable Gulf Environment, Biodiversity and Preservation: Magnes Press; 2008 p. 399–417.

[27] Mouanga GH, Langer MR. At the front of expanding ranges: Shifting community structures at amphisteginid species range margins in the Mediterranean Sea. Neues Jahrbuch für Geologie und Paläontologie—Abhandlungen. 2014;271(2):141–50. 10.1127/0077-7749/2014/0381

[28] Reinhardt EG, Patterson RT, Schroeder-Adams CJ. Geoarchaeology of the ancient harbor site of Caesarea Maritima, Israel; evidence from sedimentology and paleoecology of benthic foraminifera. J Foraminifer Res. 1994;24(1):37–48. 10.2113/gsjfr.24.1.37

[29] Yanko V, Kronfeld J, Flexer A. Response of benthic foraminifera to various pollution sources; implications for pollution monitoring. J Foraminifer Res. 1994;24(1):1–17.

[30] Hyams-Kaphzan O, Almogi-Labin A, Sivan D, Benjamini C. Benthic foraminifera assemblage change along the southeastern Mediterranean inner shelf due to fall-off of Nile-derived siliciclastics. Neues Jahrbuch Fur Geologie Und Palaontologie-Abhandlungen. 2008;248(3):315–44. 10.1127/0077-7749/2008/0248-0315 WOS:000257242500006.

[31] Arieli RN, Almogi-Labin A, Abramovich S, Herut B. The effect of thermal pollution on benthic foraminiferal assemblages in the Mediterranean shoreface adjacent to Hadera power plant (Israel). Mar Pollut Bull. 2011;62(5):1002–12. 10.1016/j.marpolbul.2011.02.036 WOS:000291133500028. 21420692

[32] Meriç E, Yokes MB, Avsar KN, Kirki-Elmas E, Dinçer F, Karhan SU, et al First report of Pararotalia calcariformata from the Hatay coastline (Turkey—north-eastern Mediterranean). Marine Biodiversity Records. 2013;6:e31.

[33] Langer MR. Epiphytic foraminifera. Mar Micropaleontol. 1993;20(3–4):235–65. 10.1016/0377-8398(93)90035-V

[34] Hyams O, Almogi-Labin A, Benjaminia C. Larger foraminifera of the southeastern Mediterranean shallow continental shelf off Israel. Isr J Earth Sci. 2002;51(3):169–79.

[35] Reiss Z, Hottinger L. Gulf of Aqaba: ecological micropaleontology. Berlin [etc.]: Springer; 1984.

[36] Parker JH, Gischler E, Eisenhauer A. Biodiversity of foraminifera from Late Pleistocene to Holocene coral reefs, South Sinai, Egypt. Mar Micropaleontol. 2012;86–87(0):59–75. 10.1016/j.marmicro.2012.02.002

[37] Madkour H. Recent benthic foraminifera of shallow marine environment from the Egyptian red sea coast. Global Advanced Research Journal of Geology and Mining Research. 2013;2(1):5–14.

[38] Hottinger L, Halicz E, Reiss Z. Recent foraminifera from the Gulf of Aqaba, Red Sea. Ljubljana, Slovenia: Slovenska Akademija Znanosti in Umetnosti; 1993.

[39] Hesemann M. *Pararotalia* sp. Accessed at http://www.foraminifera.eu/single.php?no=1003787&aktion=suche on 2014-10-22014.

[40] Goldstein ST, Alve E. Experimental assembly of foraminiferal communities from coastal propagule banks. Mar Ecol Prog Ser. 2011;437:1–11. 10.3354/meps09296 WOS:000295342600001.

[41] Reiss Z, Klug K, Merling P. Recent foraminifera from the Mediterranean and the Red Sea coasts of Israel: Bulletin of the Geological Survey of Israel, v. 32. 1961.

[42] Hyams-Kaphzan O, Grossowicz LP, Almogi-Labin A. Characteristics of benthic foraminifera inhabiting rocky reefs in northern Israeli Mediterranean shelf. Israel Geology Survey Report, GSI/36/2014, 32 p., 2014.

[43] Mancin N, Pirini C, Lanfranchini P. New species of Pararotalia Le Calvez, in Pliocene sediments of the Lower Valsesia and Western Liguria. Bolletino della Societa Palaontologica Italiana. 2000;39(3):341–50.

[44] Meric E, Avsar N, Yokes MB. Some alien foraminifers along the Aegean and southwestern coasts of Turkey. Micropaleontology. 2008;54(3–4):307–49. WOS:000261444300007.

[45] Haunold TG, Baal C, Piller WE. Benthic foraminiferal associations in the northern Bay of Safaga, Red Sea, Egypt. Mar Micropaleontol. 1997;29(3):185–210.

[46] Cedhagen T, Middelfart P. Attachment to gastropod veliger shells—a possible mechanism of disperal in benthic foraminiferans. Phuket Marine Bilological Center Special Publication. 1998;18:117–22.

[47] Wolf MA, Sfriso A, Moro I. Thermal pollution and settlement of new tropical alien species: The case of *Grateloupia yinggehaiensis* (Rhodophyta) in the Venice Lagoon. Estuarine, Coastal and Shelf Science. 2014;147(0):11–6.

[48] Bresler V, Yanko V. Chemical ecology—a new approach to the study of living benthic epiphytic Foraminifera. J Foraminifer Res. 1995;25(3):267–79. WOS:A1995RQ54700008.

[49] Bresler V, Yanko V. Acute toxicity of heavy-metals for benthic epiphytic foraminifera *Pararotalia spinigera*, Le Calvez and influence of seaweed-derived DOC. Environ Toxicol Chem. 1995;14(10):1687–95. WOS:A1995RV70900008.

[50] Loeblich AR, Tappan H. Foraminiferal genera and their classification. New York: VanNostrand Reinhold; 1987.

[51] Hottinger L, Halicz E, Reiss Z. The foraminiferal genera Pararotalia, Neorotalia, and Calcarina: taxonomic revision. J Paleontol. 1991;65(1):18–33.

[52] McCulloch I. Qualitative observations on recent foraminiferal tests with emphasis on the eastern Pacific: Parts I–III. Los Angeles, CA: University of Southern California; 1977.

[53] Hesemann M. Concept for a foraminiferal database In: Hesemann M, editor. Forams 2010, International Symposium on Foraminifera; Rheinische Friedrich-Wilhelms-Universität Bonn, 9 5–10, 2010 Germany2010.

[54] Pawlowski J, Holzmann M, Tyszka J. New supraordinal classification of foraminifera: Molecules meet morphology. Mar Micropaleontol. 2013;100:1–10. 10.1016/j.marmicro.2013.04.002 WOS:000321536600002.

[55] Schweizer M, Pawlowski J, Kouwenhoven TJ, Guiard J, van der Zwaan B. Molecular phylogeny of Rotaliida (Foraminifera) based on complete small subunit rDNA sequences. Mar Micropaleontol. 2008;66(3–4):233–46. WOS:000254134000006.

[56] Lee JJ, Anderson OR. Symbiosis in Foraminifera In: Lee JJ, Anderson OR, editors. Biology of foraminifera. San Diego: Academic Press Limited; 1991 p. 157–220.

[57] Lee JJ, Morales J, Symons A, Hallock P. Diatom symbionts in larger foraminifera from Caribbean hosts. Mar Micropaleontol. 1995;26(1–4):99–105. ISI:A1995TQ75700010.

[58] Lee JJ, Correia M. Endosymbiotic diatoms from previously unsampled habitats. Symbiosis. 2005;38:251–60. ISI:000228392500003.

[59] Lee JJ. Living Sands—The symbiosis of protists and their algae can provide good models for the study of host/symbiont interactions. Bioscience. 1995;45(4):252–61.

[60] Lee JJ, McEnery ME, Shilo M, Reiss Z. Isolation and cultivation of diatom symbionts from larger foraminifera (Protozoa). Nature. 1979;280(5717):57–8.

[61] Lee JJ. Symbiosis in foraminifera In: W R, editor. Algae and Symbioses: Biopress Ltd, Bristol, UK; 1992 p. 63–78.

[62] Lee JJ. Algal symbiosis in larger foraminifera. Symbiosis. 2006;42(2):63–75. ISI:000243459500002.

[63] Lee JJ, Reimer CW, Correia M, Morales J. A revised description of the Nitzschia frustulum var. symbiotica Lee and Reimer emend. complex, the most common of the endosymbiotic diatoms in larger foraminifera. Micropaleontology. 2000;46:170–81. ISI:000088250500014.

[64] Sarno D, Zingone A, Saggiomo V, Carrada G. Phytoplankton biomass and species composition in a Mediterranean coastal lagoon. Hydrobiologia. 1993;271(1):27–40. 10.1007/BF00005692

[65] Yilmaz P, Parfrey LW, Yarza P, Gerken J, Pruesse E, Quast C, et al The SILVA and "All-species Living Tree Project (LTP)" taxonomic frameworks. Nucleic Acids Res. 2014;42(D1):D643–D8. 10.1093/nar/gkt1209 WOS:000331139800094. 24293649 PMC3965112

[66] Ralph PJ, Gademann R. Rapid light curves: A powerful tool to assess photosynthetic activity. Aquat Bot. 2005;82(3):222–37.

[67] Ziegler M, Uthicke S. Photosynthetic plasticity of endosymbionts in larger benthic coral reef foraminifera. J Exp Mar Biol Ecol. 2011;407(1):70–80. 10.1016/j.jembe.2011.07.009 WOS:000295299200011.

[68] Nobes K, Uthicke S, Henderson R. Is light the limiting factor for the distribution of benthic symbiont bearing foraminifera on the Great Barrier Reef? J Exp Mar Biol Ecol. 2008;363(1–2):48–57. 10.1016/j.jembe.2008.06.015 ISI:000259419800007.

[69] Sinutok S, Hill R, Doblin MA, Ralph PJ. Diurnal photosynthetic response of the motile symbiotic benthic foraminiferan *Marginopora vertebralis* . Mar Ecol Prog Ser. 2013;478:127–38. WOS:000316617800010.

[70] Sunda WG, Huntsman SA. Interrelated influence of iron, light and cell size on marine phytoplankton growth. Nature. 1997;390(6658):389–92.

[71] Parkhill J-P, Maillet G, Cullen JJ. Flourescence based maximal quantum yield for PSII as a diagnositc of nutrient stress. J Phycol. 2001;37(4):517–29.

[72] Byrne M. Global change ecotoxicology: Identification of early life history bottlenecks in marine invertebrates, variable species responses and variable experimental approaches. Mar Environ Res. 2012;76(0):3–15. 10.1016/j.marenvres.2011.10.004 22154473

[73] Goldstein ST. Gametogenesis and the antiquity of reproductive pattern in the Foraminiferida. J Foraminifer Res. 1997;27(4):319–28. WOS:A1997YD22800009.

[74] Hohenegger J. Large foraminifera—greenhouse constructions and gardeners in the oceanic microcosm: The Kagoshima University Muesum, Kagoshima, Bulletin No. 5; 2011.

[75] Hohenegger J, Briguglio A, Eder W. The natural laboratory of algal symbiont-bearing benthic foraminifera: studying individual growth and population dynamics in the sublittoral In: Kitazato H, M. Bernhard J, editors. Approaches to study living foraminifera. Environmental Science and Engineering: Springer Japan; 2014 p. 13–28.

[76] Briguglio A, Hohenegger J. Growth oscillation in larger foraminifera. Paleobiology. 2014;40(3):494–509. 10.1666/13051 WOS:000336695200011. 26166912 PMC4497532

[77] Herut B, Almogi-Labin A, Jannink N, Gertman I. The seasonal dynamics of nutrient and chlorophyll a concentrations on the SE Mediterranean shelf-slope. Oceanologica Acta. 2000;23:771–82. 10.1016/s0399-1784(00)01118-x WOS:000166936600003.

[78] Alve E, Goldstein ST. Resting stage in benthic foraminiferal propagules: a key feature for dispersal? Evidence from two shallow-water species. Journal of Micropalaeontology. 2002;21(1):95–6.

[79] Tsuchiya M, Takahara K, Aizawa M, Suzuki-Kanesaki H, Toyofuku T, Kitazato H. How has foraminiferal genetic diversity developed? A case study of *Planoglabratella opercularis* and the species concept inferred from its ecology, distribution, genetics, and breeding behavior In: Kitazato H, M. Bernhard J, editors. Approaches to Study Living Foraminifera. Environmental Science and Engineering: Springer Japan; 2014 p. 133–62.

[80] Uthicke S, Vogel N, Doyle J, Schmidt C, Humphrey C. Interactive effects of climate change and eutrophication on the dinoflagellate-bearing benthic foraminifer *Marginopora vertebralis* . Coral Reefs. 2012;31(2):401–14. 10.1007/s00338-011-0851-2 WOS:000303450100011.

[81] Shaltout M, Omstedt A. Recent sea surface temperature trends and future scenarios for the Mediterranean Sea. Oceanologia. 2014;56(3):411–43. 10.5697/oc.56-3.411 WOS:000338691100001.

[82] IOLR. Statistical table for MedGLOSS stations and seasons, MedGLOSS sea level network. [Available at http://medgloss.ocean.org.il/statistic.asp.]2010.

[83] Weinmann AE, Rödder D, Lötters S, Langer MR. Traveling through time: The past, present and future biogeographic range of the invasive foraminifera *Amphistegina spp*. in the Mediterranean Sea. Mar Micropaleontol. 2013;105:30–9. 10.1016/j.marmicro.2013.10.002 WOS:000329001200003.

[84] Elshanawany R, Ibrahim MI, Milker Y, Schmiedl G, Badr N, Kholeif SEA, et al Anthropogenic impact on benthic foraminifera, Abu-Qir Bay, Alexandria, Egypt. J Foraminifer Res. 2011;41(4):326–48.

[85] Samir AM, Abdou HF, Zazou SM, El-Menhawey WH. Cluster analysis of recent benthic foraminifera from the northwestern Mediterranean coast of Egypt. Rev Micropaleontol. 2003;46(2):111–30. 10.1016/S0035-1598(03)00018-7

[86] Oczkowski A, Nixon S. Increasing nutrient concentrations and the rise and fall of a coastal fishery; a review of data from the Nile Delta, Egypt. Estuarine, Coastal and Shelf Science. 2008;77(3):309–19. 10.1016/j.ecss.2007.11.028

[87] Nixon SW. Replacing the nile: are anthropogenic nutrients providing the fertility once brought to the Mediterranean by a great river? AMBIO: A journal of the human environment. 2003;32(1):30–9. 10.1579/0044-7447-32.1.30 12691489

[88] Koukousioura O, Dimiza MD, Triantaphyllou MV, Hallock P. Living benthic foraminifera as an environmental proxy in coastal ecosystems: A case study from the Aegean Sea (Greece, NE Mediterranean). Journal of Marine Systems. 2011;88(4):489–501.

[89] Stamou AI, Kamizoulis G. Estimation of the effect of the degree of sewage treatment on the status of pollution along the coastline of the Mediterranean Sea using broad scale modelling. J Environ Manag. 2009;90(2):931–9. 10.1016/j.jenvman.2008.02.008 18406511

[90] UNEP/MAP. Initial integrated assessment of the Mediterranean Sea: fulfilling Step 3 of the ecosystem approach process 2012. UNEP(DEPI)/MED IG.20/Inf.8]. Available from: http://195.97.36.231/acrobatfiles/12IG20_Inf8_Eng.pdf

[91] Frontalini F, Kaminski M, Mikellidou I, Armynot du Châtelet E. Checklist of benthic foraminifera (class Foraminifera: d’Orbigny 1826; phylum Granuloreticulosa) from Saros Bay, northern Aegean Sea: a biodiversity hotspot. Mar Biodiv. 2014:1–19.

[92] Jueterbock A, Tyberghein L, Verbruggen H, Coyer JA, Olsen JL, Hoarau G. Climate change impact on seaweed meadow distribution in the North Atlantic rocky intertidal. Ecology and Evolution. 2013;3(5):1356–73. 10.1002/ece3.541 23762521 PMC3678489

[93] Hill R, Schreiber U, Gademann R, Larkum AWD, Kuhl M, Ralph PJ. Spatial heterogeneity of photosynthesis and the effect of temperature-induced bleaching conditions in three species of corals. Mar Biol. 2004;144(4):633–40. 10.1007/s00227-003-1226-1 ISI:000220560600002.

[94] Schmidt C, Kucera M, Uthicke S. Combined effects of warming and ocean acidification on coral reef foraminifera *Marginopora vertebralis* and *Heterostegina depressa* . Coral Reefs. 2014;33(3):805–18. 10.1007/s00338-014-1151-4

[95] SAS. JMP statistics software, Version 11. SAS; 2014.

[96] Reinhardt EG, Fitton RJ, Schwarcz HP. Isotopic (Sr, O, C) indicators of salinity and taphonomy in marginal marine systems. J Foraminifer Res. 2003;33(3):262–72. WOS:000185424000006.

[97] ter Kuile B, Erez J. In situ growth rate experiments on the symbiont-bearing foraminifera *Amphistegina lobifera* and *Amphisorus hemprichii* . J Foraminifer Res. 1984;14(4):262–76. 10.2113/gsjfr.14.4.262

[98] Medlin L, Elwood HJ, Stickel S, Sogin ML. The characterization of enzymatically amplified eukaryotic 16s-like rRNA-coding regions. Gene. 1988;71(2):491–9. 10.1016/0378-1119(88)90066-2 WOS:A1988R610900027. 3224833

[99] de Vargas C, Zaninetti L, Hilbrecht H, Pawlowski J. Phylogeny and rates of molecular evolution of planktonic foraminifera: SSU rDNA sequences compared to the fossil record. J Mol Evol. 1997;45(3):285–94. 10.1007/PL00006232 9302323

[100] Katoh K, Standley DM. MAFFT Multiple Sequence Alignment Software Version 7: Improvements in Performance and Usability. Mol Biol Evol. 2013;30(4):772–80. 10.1093/molbev/mst010 WOS:000317002300004. 23329690 PMC3603318

[101] Darriba D, Taboada GL, Doallo R, Posada D. jModelTest 2: more models, new heuristics and parallel computing. Nat Meth. 2012;9(8):772-. WOS:000307015700005. 10.1038/nmeth.2109 PMC4594756 22847109

[102] Guindon S, Dufayard JF, Lefort V, Anisimova M, Hordijk W, Gascuel O. New algorithms and methods to estimate maximum-likelihood phylogenies: assessing the performance of PhyML 3.0. Syst Biol. 2010;59(3):307–21. 10.1093/sysbio/syq010 WOS:000276528300006. 20525638

[103] Letunic I, Bork P. Interactive Tree Of Life v2: online annotation and display of phylogenetic trees made easy. Nucleic Acids Res. 2011;39:W475–W8. 10.1093/nar/gkr201 WOS:000292325300077. 21470960 PMC3125724

[104] Pruesse E, Peplies J, Glockner FO. SINA: Accurate high-throughput multiple sequence alignment of ribosomal RNA genes. Bioinformatics. 2012;28(14):1823–9. 10.1093/bioinformatics/bts252 WOS:000306136100004. 22556368 PMC3389763

[105] Tyberghein L, Verbruggen H, Pauly K, Troupin C, Mineur F, De Clerck O. Bio-ORACLE: a global environmental dataset for marine species distribution modelling. Glob Ecol Biogeogr. 2012;21(2):272–81. 10.1111/j.1466-8238.2011.00656.x WOS:000298912900016.

[106] Langer MR, Weinmann AE, Lötters S, Bernhard JM, Rödder D. Climate-driven range extension of Amphistegina (Protista, Foraminiferida): models of current and predicted future ranges. PLoS ONE. 2013;8:e54443 10.1371/journal.pone.0054443 23405081 PMC3566174

[107] Weinmann AE, Rödder D, Lötters S, Langer MR. Heading for new shores: projecting marine distribution ranges of selected larger foraminifera. PLoS ONE. 2013;8:e62182 10.1371/journal.pone.0062182 WOS:000317909500090. 23620810 PMC3631173

[108] Phillips SJ, Anderson RP, Schapire RE. Maximum entropy modeling of species geographic distributions. Ecol Model. 2006;190(3–4):231–59. 10.1016/j.ecolmodel.2005.03.026 WOS:000233859600001.

[109] Jaynes ET. Information theory and statistical mechanics. Physical Review. 1957;106(4):620–30. 10.1103/PhysRev.106.620 WOS:A1957WB72300004.

[110] Elith J, Phillips SJ, Hastie T, Dudik M, Chee YE, Yates CJ. A statistical explanation of MaxEnt for ecologists. Divers Distrib. 2011;17(1):43–57. 10.1111/j.1472-4642.2010.00725.x WOS:000285246700005.

[111] Phillips SJ, Dudik M. Modeling of species distributions with Maxent: new extensions and a comprehensive evaluation. Ecography. 2008;31(2):161–75. 10.1111/j.0906-7590.2008.5203.x WOS:000254499200001.

[112] Liu CR, Berry PM, Dawson TP, Pearson RG. Selecting thresholds of occurrence in the prediction of species distributions. Ecography. 2005;28(3):385–93. 10.1111/j.0906-7590.2005.03957.x WOS:000229428800011.

[113] Locarnini RA, Mishonov, A.V., Antonov, J.I., Boyer, T.P., Garcia, H.E. NOAA Atlas NESDIS 61. World Ocean Atlas 2005, Volume 1: Temperature. S. Levitus. U.S. Government. 2006.

