# Supplementary material for: Recent Invasion of the Symbiont-Bearing Foraminifera Pararotalia into the Eastern Mediterranean Facilitated by the Ongoing Warming Trend
Source: PLoS One. 2015 Aug 13;10(8):e0132917. doi: 10.1371/journal.pone.0132917 (PMC4536047; doi:10.1371/journal.pone.0132917)
Supplement: S1 File — This file includes a documentation of shell morphology of late ontogenetic stages and their specific features (Plate A) and juvenile from field collections (early ontogenetic stages, Plate B) in Nachscholim National Park, Israel. (DOCX) [file pone.0132917.s001.docx]

**Taxonomic description and documentation of morphology of the invasive Mediterranean *Pararotalia calcariformata***

Superfamily ROTALIOIDEA Ehrenberg, 1839

Family ROTALIIDAE Ehrenberg, 1839

Subfamily PARAROTALIINAE Reiss, 1963

Genus *Pararotalia* Le Calvez, 1949

*Pararotalia calcariformata* McCulloch, 1977

Not *Pararotalia* *spinigera* Le Calvez, 1949, emend. by [Loeblich and Tappan (1957](#_ENREF_4)), Plate 4, Figs 1-3, [middle Eocene, Lutetian, Calcarie Grossier, France]

Not *Pararotalia* *spinigera* Le Calvez, 1949, [Hottinger et al. (1991](#_ENREF_2)) Plate 1 Figs. 2, 5-8 [middle Eocene, Lutetian, Grignon, Paris Basin, France]

*Pararotalia* (?) *calcariformata* McCulloch, 1977, [McCulloch (1977](#_ENREF_6)), Plate 177, Figs 10-11, [Recent, type locality Colombo Bay, shallow waters, Station 616, off West Nole Island, Australia, shallow waters]

*Pararotalia calcariformata* McCulloch 1977, [Loeblich and Tappan (1994](#_ENREF_5)), Plate 367, Figs. 10-13, [Hypotype, Southern Timor Sea, Figs 11-13 sample V-347 at 58.52 m, Recent, North of Bathurst Island, southeast Timor Sea, 1961 Sahul Shelf Cruise II Stranger]

*Pararotalia* *spinigera* Le Calvez 1949, [Reinhardt et al. (1994](#_ENREF_8)), Plate 2, Figs 11-12. [Late Holocene, Recent, CT/Grab 3, 9 m, Caesarea, Israel]

*Eponides repandus* Fichtel et Moll, 1798, [Yanko et al. (1994](#_ENREF_9)), Plate 2, Figs. 1-9 [Recent, Haifa Bay, Station 27, 12 meters]

*Pararotalia* *spinigera* Le Calvez, 1949, [Arieli et al. (2011](#_ENREF_1)), Plate 6, Figs. 9-12 [Recent, Hadera, Israel]

*Pararotalia* (?) *calcariformata* McCulloch, 1977, [Meriç et al. (2013](#_ENREF_7)), Plate 1 Figs. 1-11, not 12 [Recent, Hatay, Turkey, 3-8 meters]

**Original species description:** Test free, calcareous, auriculate, biconvex, trochospiral, periphery lobulated, craniate with usually one short spine per chamber, dorsally wall rather smooth semi-hyaline, finely perforate; dorsal side evolute showing less than three whorls of slightly inflated subrhomboidal chambers gradually increasing in size; sutures dorsally limbate darker hyaline flush to depressed curved bands. Ventral side involute with prominent umbilical, raised nodulous, hyaline to umbilical plug surrounded by nodulous umbilical flaps, usually seven chambers in the last formed whorl; keel shallow hyaline acute with most of the tapering peripheral chambers forming a single short angular hyaline spine extending out in posteriorly from anterior half of each chamber of last formed whorl; sutures ventrally rather broad limbate depressed radiate; aperture interiomarginal extraumbilical umbilical with a narrow depressed rim outlining a low arch beginning at the close to the periphery, no tooth plate visible due to excess shell on most specimens.

**Comments**: The Levantine *Pararotalia* examined by us (see Plate 1 and 2) reveals a significant morphological variability, which is accentuated by ontogeny. Juvenile forms (Plate 2) typically possess trapezoidal chambers and single pseudospines (occasionally two pseudospines per chamber) extending from most chambers of the last whorl, occasionally positioned near the septal face. Chambers of the last whorl in fully developed adults are usually petaloid and lack the peripheral pseudospines. The peripheral margins and keel of the umbilical side in both juveniles and adults are covered with numerous short pustules, found also in *P.* *spinigera* Le Calvez 1949. The studied specimens from Israel reveal two pore structures that have not been documented previously: micropores and larger circular to elliptical pore mounds on the surface of both sides of the test in juvenile and adult forms. The chamber wall of *Pararotalia sp.* is highly transparent, similar to other foraminifera known to host symbionts such as other Calcarinids. It has been suggested that such a wall structure can be seen as an adaptation to photosymbiosis ([Leutenegger 1984](#_ENREF_3)). The range of variability in the examined populations include all morphologies figured by [Meriç et al. (2013](#_ENREF_7)). These authors note the lack of spines in their specimens, but we believe they do so in contrast to species of *Pararotalia* with more prominent spines, such as *P. stellata*. Not all of the Levantine specimens studied by us had spines (Plate 1, 1-3). Also, we note that a specimen pictured by [Meriç et al. (2013](#_ENREF_7)) in their Figure 2, image 4a, seems to possess spines of a similar shape and extent as in the studied population from Israel (Plate 1, 4). Therefore, we conclude that the populations from Israel and Hatay, Turkey, are morphologically overlapping. Following [Meriç et al. (2013](#_ENREF_7)) we conclude that the Levantine *Pararotalia* is morphologically distinct from the concept of *P. spinigera*. The latter species has a lobate periphery without keel, which is a distinct and persistent feature of the Levantine population (Plate 1, 2-4, 6). In addition, in the Levantine population the umbilical sutures are so deeply incised that the walls of adjacent chambers become partly disconnected, forming deep interlocular spaces (Plate 1, 1, 3, 4, 6). This character is indicated in the illustration of the type material of *P. calcariformata* ([McCulloch 1977](#_ENREF_6)). The particular character also clearly distinguishes this species from the youngest fossil species of the genus in the Mediterranean, described by Mancin et al. (2000) from Pliocene sediments in northern Italy as *Pararotalia padana*. The Pliocene *P. padana* also show considerable variability, similar overall shape and presence of spines. On the other hand, *P. padana* has more chambers in the last whorl, more lobate periphery and completely lacks the interlocular spaces between successive chambers that are observed on the umbilical side of many specimens *in P. calcariformata*. In addition, *P. padana* does not show the distinct depressed area near the periphery on the umbilical side of the shell (see specimen in Plate 1, Figure 3d) and appears to lack well developed nodes that surround the chambers of *P. calcariformata* on the umbilical side.

**Plate A**

**Late ontogenetic stages of *Pararotalia calcariformata***

**1.** Adult specimen with dextral coiling. **a-b** spiral views, note the slightly raised sutures, a rounded peripheral outline, the spinose peripheral keel, and the central thickening of the early whorls. **b**. Details showing densely perforated pore fields between imperforate septal sutures. **c-d** Umbilical (ventral) views, note the interlocular space between consecutive chambers, the presence of umbilical plug fused with the umbilical wall of the surrounding chambers, the thick marginal nodes on early chambers, and the hirsute peripheral surface with numerous pustules.

**2.** **a.** Spiral view of fully developed adult specimen (sinistral coiling), with nine chambers in the last whorl. **b-c** Lateral view of adult specimen showing interiomarginal aperture

**3.** Adult specimen with dextral coiling. **a-b** Spiral view with a frequent well-developed pore mounds. **c-d** Umbilical view showing umbilical plug, large nodes along the edges of the sutures and chamber surface with well-developed pore mounds. **e.** Lateral view showing interiomarginal aperture.

**4.** Adult specimen with dextral coiling. **a-b** Spiral views showing nodose ornamentation, peripheral spines on early chambers, and microperforate surface of the ultimate chamber. **c-d** Umbilical views showing details of a peripheral spine. **e** Lateral view showing interiomarginal aperture and keeled outline.

**5.** Adult specimen with sinistral coiling. **a-c** Spiral side, note the early nodose whorls and the microperforate surface of the ultimate chamber. **d** Umbilical view showing strong nodose ornamentation.

**6.** Adult dextral coiled specimen with peripheral spines. **a** Spiral view. **b** Umblical view.

**c** Lateral view.

Scale Bars: 100 µm

**
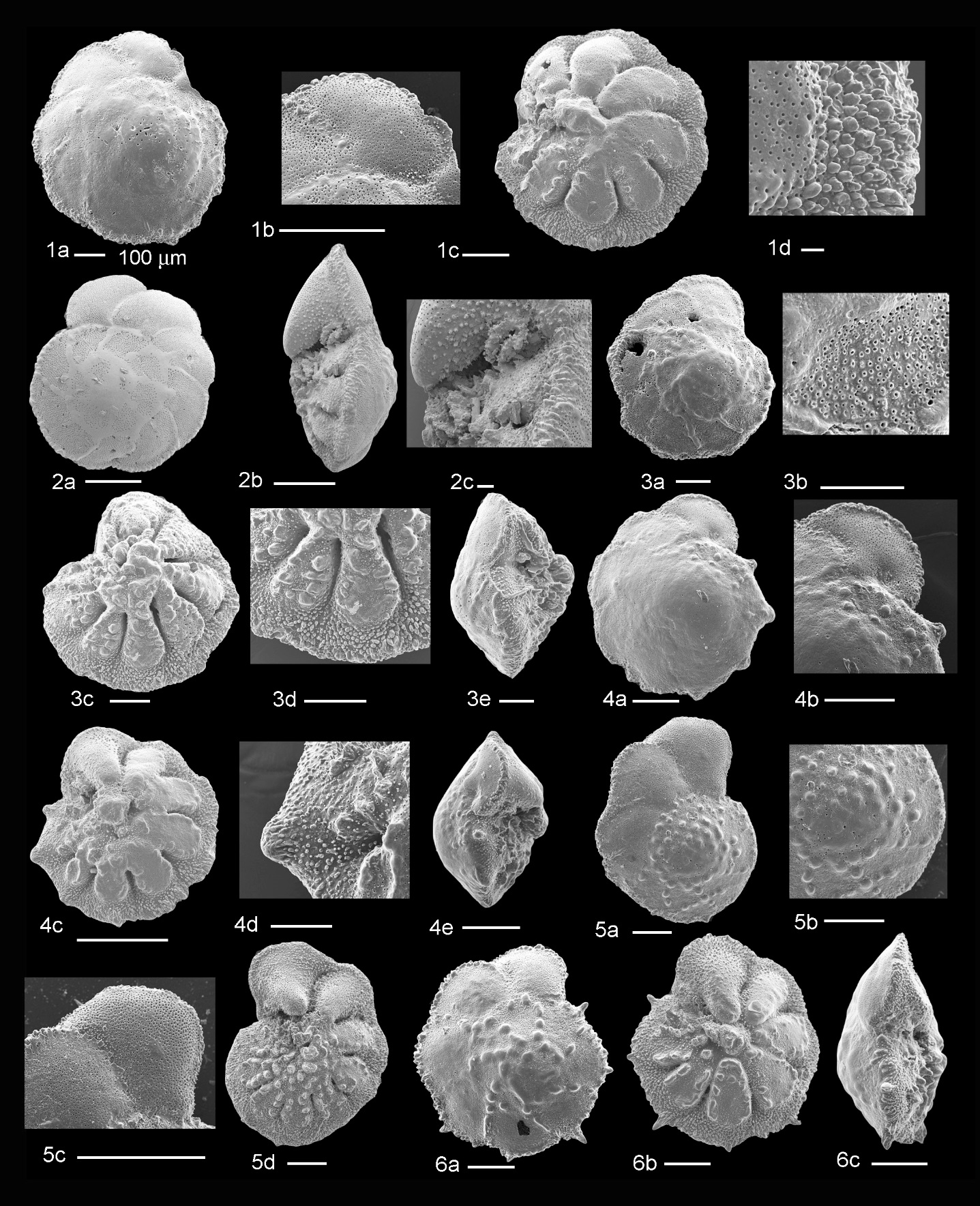
**

**Plate B**

**Early ontogenetic stages of *Pararotalia calcariformata***

**1.-3.** Earliest stage specimens with three and four chambers. **1** Spiral view of specimen with incipient peripheral pseudospines. **2** Spiral view of specimen with peripheral pseudospines. **3**. Umbilical view of specimens without peripheral pseudospines, showing incipient keel. Note the pustulate surface.

**4.** Juvenile specimen with peripheral pseudospine on each chamber. **a**. Spiral view.

**b** Umbilical view, note the distinct trapezoidal shape of the chambers in the spiral and umbilical sides. **c** Lateral view. **d** Details of the interiomarginal aperture.

**5.** Juvenile specimen with peripheral pseudospine on each chamber. **a- b** Spiral views showing well-developed pore mounds and trapezoidal shaped chambers.

**6.** Juvenile specimen with peripheral pseudospine on each chamber. **a- c** Spiral views. **d-f** Umbilical views, note the relatively large pore mounds on the surface both sides of the test. **f** Details of the peripheral pseudospine.

Scale Bars: 1-3 = 10µm, 4-6d = 100µm and 6e = 50µm


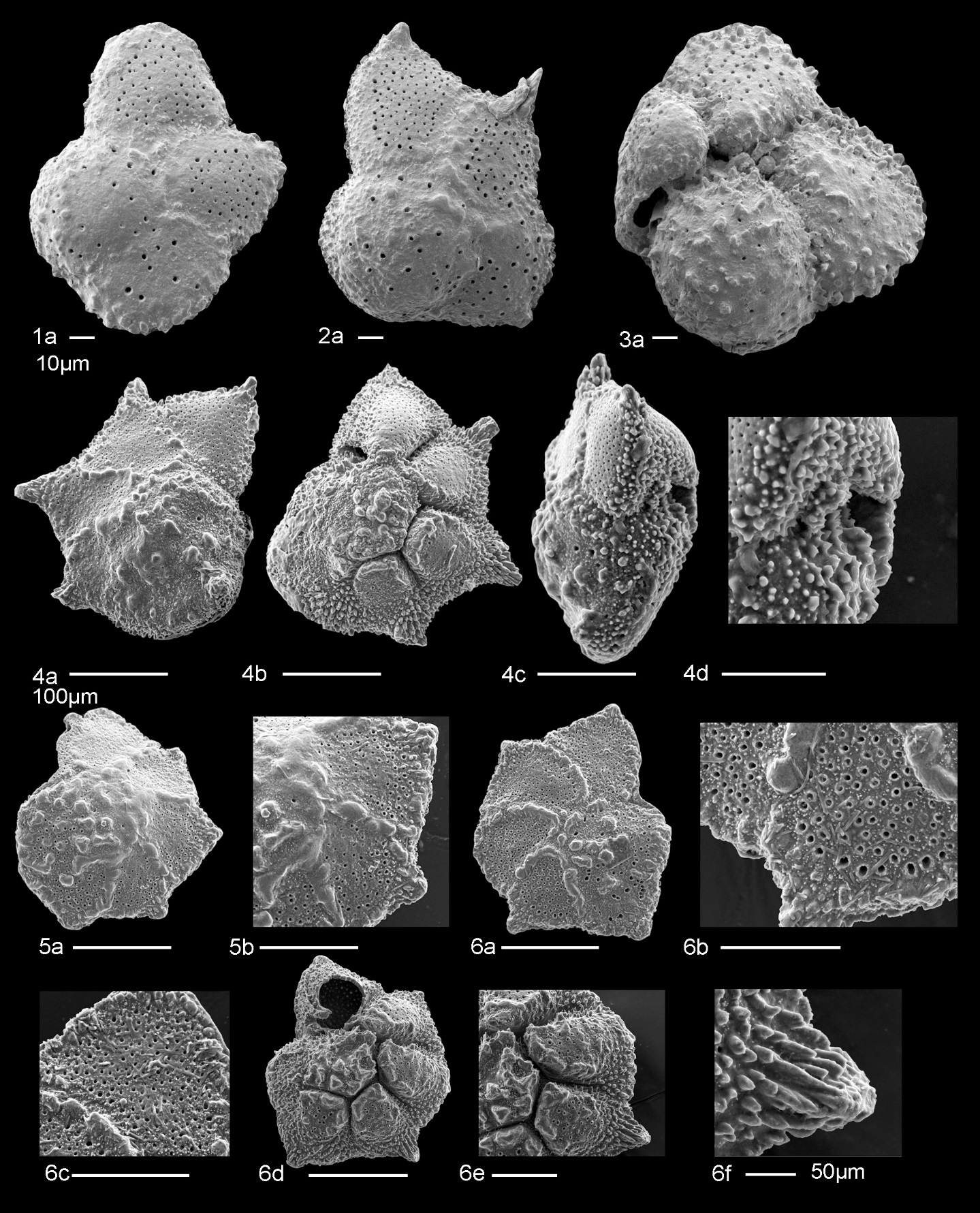


References:

Arieli RN, Almogi-Labin A, Abramovich S, Herut B (2011) The effect of thermal pollution on benthic foraminiferal assemblages in the Mediterranean shoreface adjacent to Hadera power plant (Israel). Mar Pollut Bull 62:1002-1012

Hottinger L, Halicz E, Reiss Z (1991) The Foraminiferal Genera Pararotalia, Neorotalia, and Calcarina: Taxonomic Revision. J Paleontol 65:18-33

Leutenegger S (1984) Symbiosis in benthic foraminifera; specificity and host adaptations. J Foraminifer Res 14:16-35

Loeblich A, Tappan H (1957) Eleven new genera of Foraminifera. US National Museum Bulletin 215 223-232

Loeblich AR, Tappan H (1994) Foraminifera of the Sahul Shelf and Timor Sea. J Foraminifer Res Special Publications 31

Mancin N, Pirini C, Lanfranchini PL (2000) New species of *Pararotalia* Le Calvez, in Pliocene sediments of the Lower Valsesia and Western Liguria. Bollettino della Socitá Paleontologica Italiana 39(3): 341-350

McCulloch I (1977) Qualitative observations on recent foraminiferal tests with emphasis on the eastern Pacific: Parts I–III. University of Southern California, Los Angeles, CA

Meriç E, Yokes MB, Avsar KN, Kirki-Elmas E, Dinçer F, Karhan SU, Kalkan E, Demir V (2013) First report of Pararotalia calcariformata from the Hatay coastline (Turkey—north-eastern Mediterranean). Marine Biodiversity Records 6:e31

Reinhardt EG, Patterson RT, Schroeder-Adams CJ (1994) Geoarchaeology of the ancient harbor site of Caesarea Maritima, Israel; evidence from sedimentology and paleoecology of benthic foraminifera. J Foraminifer Res 24:37-48

Yanko V, Kronfeld J, Flexer A (1994) Response of benthic Foraminifera to various pollution sources; implications for pollution monitoring. J Foraminifer Res 24:1-17
